# Comparative analysis and implications of the chloroplast genomes of three thistles (*Carduus* L., Asteraceae)

**DOI:** 10.7717/peerj.10687

**Published:** 2021-01-14

**Authors:** Joonhyung Jung, Hoang Dang Khoa Do, JongYoung Hyun, Changkyun Kim, Joo-Hwan Kim

**Affiliations:** 1 Department of Life Science, Gachon University, Seongnam, Gyeonggi, Korea; 2 Nguyen Tat Thanh Hi-Tech Institute, Nguyen Tat Thanh University, Ho Chi Minh City, Vietnam

**Keywords:** *Carduus crispus*, Chloroplast genome, Invasive species, Molecular markers, Plumeless thistles

## Abstract

**Background:**

*Carduus*, commonly known as plumeless thistles, is a genus in the Asteraceae family that exhibits both medicinal value and invasive tendencies. However, the genomic data of *Carduus* (i.e., complete chloroplast genomes) have not been sequenced.

**Methods:**

We sequenced and assembled the chloroplast genome (cpDNA) sequences of three *Carduus* species using the Illumina Miseq sequencing system and Geneious Prime. Phylogenetic relationships between *Carduus* and related taxa were reconstructed using Maximum Likelihood and Bayesian Inference analyses. In addition, we used a single nucleotide polymorphism (SNP) in the protein coding region of the *matK* gene to develop molecular markers to distinguish *C. crispus* from *C. acanthoides* and *C. tenuiflorus*.

**Results:**

The cpDNA sequences of *C. crispus, C. acanthoides*, and *C. tenuiflorus* ranged from 152,342 bp to 152,617 bp in length. Comparative genomic analysis revealed high conservation in terms of gene content (including 80 protein-coding, 30 tRNA, and four rRNA genes) and gene order within the three focal species and members of subfamily Carduoideae. Despite their high similarity, the three species differed with respect to the number and content of repeats in the chloroplast genome. Additionally, eight hotspot regions, including *psbI-trnS_GCU*, *trnE_UUC-rpoB*, *trnR_UCU-trnG_UCC*, *psbC-trnS_UGA*, *trnT_UGU-trnL_UAA*, *psbT-psbN*, *petD-rpoA*, and *rpl16-rps3*, were identified in the study species. Phylogenetic analyses inferred from 78 protein-coding and non-coding regions indicated that *Carduus* is polyphyletic, suggesting the need for additional studies to reconstruct relationships between thistles and related taxa. Based on a SNP in *matK*, we successfully developed a molecular marker and protocol for distinguishing *C. crispus* from the other two focal species. Our study provides preliminary chloroplast genome data for further studies on plastid genome evolution, phylogeny, and development of species-level markers in *Carduus*.

## Introduction

*Carduus* L. (subfamily Carduoideae; Asteraceae), commonly known as plumeless thistles, comprises 90 species native to Eurasia and Africa (Angiosperm Phylogeny Group, 2016). Several *Carduus* species are invasive, noxious weeds on other continents (Doing, Biddiscombe & Knedlhans, 1969). Four species, including *C. acanthoides* L. (spiny plumeless thistle), *C. tenuiflorus* Curtis (sheep thistle), *C. pycnocephalus* L. (Italian thistle), and *C. crispus* Guirão ex Nyman (welted thistle), all of which originate in Eurasia and Africa, are considered invasive in North America (Dunn, 1976; Verloove, 2014). *Carduus crispus*, also called curly plumeless thistle, is also considered an invasive species in Korea (Jung et al., 2017). This species differs from other *Carduus* in having soft, sparsely arachnoid-hairy leaves with short marginal bristles, and apically recurved involucral bracts (Todorov et al., 2018). Among *Carduus* species, *C. crispus* contains chemicals with the potential to treat various diseases (Xie, Li & Jia, 2005; Davaakhuu, Sukhdolgor & Gereltu, 2010; Lee et al., 2011; Tunsag, Davaakhuu & Batsuren, 2011). Specifically, certain compounds extracted from *C. crispus* have potential value in the treatment of obesity and cancer (Davaakhuu, Sukhdolgor & Gereltu, 2010; Lee et al., 2011). While *Carduus* has been studied from various perspectives (i.e., invasion, phylogeny, and medicinal effects), its chloroplast genome has not been sequenced. It is therefore worthwhile to study the genome of *Carduus*, and particularly that of *C. crispus*, which has potential medicinal benefits.

In most angiosperms, the chloroplast genome (cpDNA) contains genes essential to photosynthesis (Sugiura, 1992). Genomic events (i.e., gene deletion, inversion, or duplication) in cpDNA may provide information about species’ evolutionary history (Cosner, Raubeson & Jansen, 2004; Do & Kim, 2017; Haberle et al., 2008). For example, the Fabaceae includes clades that are characterised by large inversions and the loss of inverted repeat regions (Choi & Choi, 2017). Inversions have also been recorded in the cpDNA of Asteraceae (Kim, Choi & Jansen, 2005). Specifically, a large inversion comprising a 22.8 kb sequence occurred simultaneously with a small inversion of a 3.3 kb fragment; this event coincided with the split between major clades (excluding Barnadesioideae) in the evolution of Asteraceae. cpDNA data can also be used to develop molecular markers based on nucleotide polymorphisms (i.e., single nucleotide polymorphism (SNP) markers and microsatellite markers). Molecular authentication has been reported for various plant species, with a focus on invasive plants, endangered species, and taxa with potential medicinal value (Kim et al., 2012; Ishikawa, Sakaguchi & Ito, 2016; Luo et al., 2016; Park et al., 2016; Marochio et al., 2017; Han et al., 2018; Do et al., 2019). Specific regions of cpDNA have been identified for developing molecular markers in plants, including the commonly-used *matK* region (Poovitha et al., 2016; Vu et al., 2017). Among the Asteraceae, studies on molecular markers have been conducted for rubber dandelion (*Taraxacum kok-saghyz* LE Rodin), horseweed (*Conyza* sp.), Indian Chrysanthemum (*Chrysanthemum indicum* L.), the endemic herb *Aster savatieri* Makino, and the invasive plant *Tithonia diversifolia* (Hemsl.) A Gray (Ishikawa, Sakaguchi & Ito, 2016; Luo et al., 2016; Zhang et al., 2017; Marochio et al., 2017; Han et al., 2018). In addition to the development of these molecular markers, complete cpDNA sequences have been reported for various Asteraceae species (Kim, Choi & Jansen, 2005; Choi & Park, 2015; Wang et al., 2015a; Wang et al., 2015b; Yun, Gil & Kim, 2017; Liu et al., 2018; Ma, Sun & Zhao, 2018; Su et al., 2018). cpDNA sequences may be used to elucidate the phylogeny of angiosperms from the clade that is basal to monocots and eudicots (Angiosperm Phylogeny Group, 2016). Previous investigations into phylogenetic relationships among members of the Asteraceae have been conducted using a range of molecular data types, including *rbcL*, *ndhF*, *matK*, chloroplast DNA restriction sites, ITS sequence data, and nuclear loci (Jansen, Michaels & Palmer, 1991; Häffner & Hellwig, 1999; Fu et al., 2016; Mandel et al., 2019). However, the paucity of available sequence data may have resulted in ambiguous relationships between *Carduus* and related taxa (Häffner & Hellwig, 1999; Fu et al., 2016). In particular, ITS sequence data suggest that *C. leptacanthus* is sister to *Cirsium* and *Notobasis*, whereas another *Carduus* species is sister to *Cirsium* and *Tyrimmus* (Häffner & Hellwig, 1999). As such, clarification of relationships between *Carduus* and related species will require studies that include a larger number of *Carduus* species and different data types (i.e., chloroplast and mitochondrial genomes).

We used next-generation sequencing (NGS) to sequence and characterise the chloroplast genomes of *Carduus crispus*, *C. acanthoides*, and *C. tenuiflorus*, which exhibit both invasive tendencies and potential medical utility (particularly *C. crispus*). We then conducted comparative genomic analyses to explore genomic diversity among the three species with respect to highly variable regions, and the types and numbers of repeats. In addition, we reconstructed the formerly ambiguous relationship between *Carduus* and related taxa based on 78 protein-coding regions and non-coding sequences. Finally, we developed a specific molecular marker for *C. crispus* based on a SNP in the *matK* gene. This molecular marker provides useful information for managing *C. crispus* invasions, particularly with respect to the identification of immature (vegetative) individuals, which tend to be morphologically similar to other *Carduus* species (i.e., having winged stems with apical spines and spiny leaves). This molecular marker may also support positive identification of *C. crispus* for medical usage.

## Materials & Methods

### Taxon sampling, total DNA extraction, chloroplast genome assembly, and comparative analysis

**Table 1:** List of *Carduus* species for NGS and developing molecular marker.

**No.**	**Species**	**Voucher**	**Location**
1	*Carduus crispus* Guirão ex Nyman	Korea National Arboretum (LK0908)	Mt. Seokbyeong, Imgye-myeon, Jeongseon-gun, Gangwon-do, Republic of Korea
2	*Carduus crispus* Guirão ex Nyman*	Korea National Arboretum (LK0943)	Mt. cheongog, Hajang-myeon, Samcheok-si, Gangwon-do, Republic of Korea
3	*Carduus crispus* Guirão ex Nyman	Korea National Arboretum (LK1497)	Mt. Nochu, Yeoryang-myeon, Jeongseon-gun, Gangwon-do, Republic of Korea
4	*Carduus crispus* Guirão ex Nyman	Korea National Arboretum (CNUFR0470)	295, Sinseong-ri, Bukha-myeon, Jangseong-gun, Jeollanam-do, Republic of Korea
5	*Carduus crispus* Guirão ex Nyman	Korea National Arboretum (LK0430)	Mt. cheongog, Imgye-myeon, Jeongseon-gun, Gangwon-do, Republic of Korea
6	*Carduus crispus* Guirão ex Nyman	National Institute of Biological Resources (NIBRVP0000619716)	Mt. Jaam, Namhu-myeon, Andong-si, Gyeongsangbuk-do, Republic of Korea
7	*Carduus crispus* Guirão ex Nyman	National Institute of Biological Resources (NIBRVP0000601328)	Sanghwa-ri, Danchon-myeon, Uiseong-gun, Gyeongsangbuk-do, Republic of Korea
8	*Carduus crispus* Guirão ex Nyman	National Institute of Biological Resources (NIBRVP0000524207)	Mt. Beophwa, Yugu-eup, Gongju-si, Chungcheongnam-do, Republic of Korea
9	*Carduus crispus* Guirão ex Nyman	National Institute of Biological Resources (NIBRVP0000613325)	Mt. Mani, Hwado-myeon, Ganghwa-gun, Incheon, Republic of Korea
10	*Carduus crispus* Guirão ex Nyman	National Institute of Biological Resources (NIBRVP0000580633)	Mt. Jeonggwang, Mohyeon-eup, Cheoin-gu, Yongin-si, Gyeonggi-do, Republic of Korea
11	*Carduus crispus* Guirão ex Nyman	National Institute of Biological Resources (NIBRVP0000578538)	Mt. Jangam, Pyeongchang-eup, Pyeongchang-gun, Gangwon-do, Republic of Korea
12	*Carduus crispus* Guirão ex Nyman	National Institute of Biological Resources (NIBRVP0000580501)	Mt. Gwangdeok, Dongnam-gu, Cheonan-si, Chungcheongnam-do, Republic of Korea
13	*Carduus acanthoides* L.*	Kim 2018-001, Nevada city, Ca, USA	USA, California, Nevada city
14	*Carduus acanthoides* L.	NewYork Botanical Garden (00532263)	USA, Colorado, Pitkin Co., South side of State Highway 82, 5 miles W of Aspen, Airport., 2256 - 2256m
15	*Carduus acanthoides* L.	NewYork Botanical Garden (00532244)	USA, Wisconsin, Richland Co., 3 miles SE of Richland Center., 43.300443 -90.321019
16	*Carduus acanthoides* L.	NewYork Botanical Garden (00532262)	USA, Wyoming, Platte Co., 1402 - 1402m
17	*Carduus acanthoides* L.	Carnegie Museum Herbarium (267884)	Romania, Oltenia, distr. Dolj, inter vicos Lascar Catargiu et Popoveni ad Canalul colector, 75m
18	*Carduus acanthoides* L.	Carnegie Museum Herbarium (528144)	USA, Pennsylvania, Mifflin, Maple & Walnut Sts, Belleville
19	*Carduus acanthoides* L.	United States National Herbarium (1944312)	Czech Republic, Bohemia centralis: Paraha-Troja. In ruderatis
20	*Carduus acanthoides* L.	United States National Herbarium (3419733)	Ukraine, Prov. Czerkassy, prope opp. Umanj, in ruderatis
21	*Carduus tenuiflorus* Curtis*	Kim 2018-002, Nevada city, Ca, USA	USA, California, Nevada city
22	*Carduus tenuiflorus* Curtis	NewYork Botanical Garden (00366662)	Mexico, El Cercado. Santiago, N. L., 495–495 m
23	*Carduus tenuiflorus* Curtis	Carnegie Museum Herbarium (519240)	USA, California, Humboldt, Angels Ranch, toward Hungry Hollow, Bald Mountain
24	*Carduus tenuiflorus* Curtis	Carnegie Museum Herbarium (282243)	USA, California, Alameda, Berkeley

**Notes.**

Asterisks indicate samples for next generation sequencing (NGS) analysis.

Leaves of *C. crispus*, *C. acanthoides*, and *C. tenuiflorus* were collected and dried in silica gel powder for NGS analysis. In addition, to test the efficiency of molecular markers (Table 1), leaves of 22 individuals of the three species were sampled from herbaria at the Korea National Arboretum (KNA), National Institute of Biological Resources (NIBR), New York Botanical Garden (NYBG), Carnegie Museum Herbarium (CM), and the United States National Herbarium (US). A modified cetyl trimethylammonium bromide (CTAB) protocol was used to extract total DNA from collected samples (Doyle & Doyle, 1987). High-quality DNA was used to conduct NGS using the Miseq platform and a Miseq Reagent Kit v3 (Illumina, Seoul, South Korea). Raw NGS data (2 × 300 bp paired-end reads) were cleaned by cutting adapter sequences, removing duplicate and chimeric reads, and trimming ends with > 0.05 probability of error per base. Cleaning was conducted using Geneious Prime (Kearse et al., 2012). Raw data were submitted to NCBI (accession number PRJNA645567). Using Geneious Prime, filtered NGS data for each species were mapped to the reference chloroplast genome sequences of *Arctium lappa* (NCBI accession number MH375874), *Saussurea polylepis* (MF695711), *Carthamus tinctorius* (KP404628), *Centaurea diffusa* (KJ690264), *Silybum marianum* (KT267161), *Cirsium arvense* (KY562583), *Cynara humilis* (KP299292), and *Atractylodes chinensis* (MG874805) to isolate cpDNA reads of which the similarity to reference was over 95%. Isolated cpDNA reads were then assembled using the de novo function in Geneious to create various contigs of chloroplast genome sequences. The newly created contigs were de novo re-assembled to construct complete cpDNA sequences for each focal species. We confirmed the results from Geneious Prime by reconstructing the complete cpDNA of *Carduus* using NOVOplasty and following Dierckxsens et al. (2017). Using Geneious Prime, gene content and order of sequenced cpDNA were annotated based on existing complete cpDNA sequences of other Asteraceae taxa. Annotations that had over 95% similarity in comparison with references were retained, and the start and stop codons in the protein coding regions were verified (Data S1). tRNA sequences were assessed using tRNA Scan-SE (Chan & Lowe, 2019). Complete cpDNA sequences of the three species were submitted to GenBank; accession numbers were MK652229 for *C. crispus*, MK652228 for *C. acanthoides*, and MK652230 for *C. tenuiflorus.* The cpDNA map was visualised using OGDraw (Greiner, Lehwark & Bock, 2019). Complete chloroplast genomes of *Carduus* species were aligned with other Asteraceae and related species (*Nicotiana tabacum* (NC_001879) was used as an outgroup; Table S1), and gene loss and rearrangement were identified using MAUVE (Darling, Mau & Perna, 2010). In addition, Geneious Prime was used to calculate the pairwise identities of cpDNA sequences in the focal species. Small single repeats (SSRs) were analysed using Phobos embedded in Geneious Prime (Christoph, 2006–2010). Minimum repeat numbers were 10, 5, 4, and 3 for mono-, di-, tri-, and tetra-nucleotides, respectively. REPuter (Kurtz et al., 2001) was used to analyse the large repeat sequences (minimum length = 20 bp) in each species. To explore nucleotide diversity, 131 coding and non-coding regions were extracted from the complete cpDNA (Table S2). Following alignment using MUSCLE embedded in Geneious Prime (Edgar, 2004), aligned sequences were imported into DnaSP 6 (Rozas et al., 2017) for calculation of Pi values.

### Phylogenetic analysis of *Carduus* and related taxa

A total of 78 protein-coding regions were extracted from the complete cpDNA of the focal species and other related taxa (Table S1). Sequences were aligned using MUSCLE embedded in Geneious Prime (Edgar, 2004). We used jModeltest (Posada, 2008) to find the best model for the aligned DNA sequences; GTR + I + R was selected as the most suitable model and was used in Maximum Likelihood (ML) and Bayesian Inference (BI) analyses. The ML analysis was conducted with the IQ-tree web server (http://iqtree.cibiv.univie.ac.at), using 1,000 bootstrap replications to calculate branch support values (Trifinopoulos et al., 2016). We used MrBayes v3.2 (Ronquist et al., 2012) for BI analyses. The Markov chain Monte Carlo (MCMC) analysis was run for 1,000,000 generations, and a tree was assembled every 1000 generations. A 25% burn-in setting was used for summarising trees. Figtree v4.0 (http://tree.bio.ed.ac.uk/software/figtree/) was used to visualise phylogenetic trees. Other datasets, including whole chloroplast genomes (excluding one IR region), non-coding regions of cpDNA, and hotspot regions derived from the cpDNA of *Carduus* species, were used in phylogenetic analysis in addition to protein-coding regions (Table S1). Analytical procedures for these additional datasets were identical to those used for the protein coding regions; however, we used the TVM+I+G model for the whole chloroplast genome (excluding one inverted repeat [IR] region) and all non-coding regions, and the TVM+G model for the hotspot regions dataset.

### SNP identification, primer design, and multiplex PCR

The complete *matK* gene, extracted from the cpDNA of the three focal species, was aligned using MUSCLE to identify SNPs (Edgar, 2004; Fig. S1). The selected SNP for *C. crispus* was then confirmed by aligning the available *matK* sequences of other *Carduus* species on NCBI to those of the focal species (Fig. S2). Based on SNP data, primer pairs were designed using Primer3 to distinguish *C. crispus* from other *Carduus* species (Untergasser et al., 2012). Primer sequences included matK_463F (5′-CATCTGGAAATCTTGGTTCAG-3′), matK_1162R (5′-GATGCCCCAATGCGTTACAA-3′), CD_SNP_F1 (5′-AATTCTTGCTTCAAAAGG GTCC- 3′), CD_SNP_R1 (5′-TTCCATTTATTCATCAA AAGATAC-3′), CD_SNP_F2 (5′-AATTCTTGCTTCAAAAGGGTCG-3′), and CD_SNP_R2 (5′-TTCCATTTATTCATCAAAAGATAG- 3′). The multiplex PCR of matK_463F, matK_1162R, CD_SNP_F1, and CD_SNP_R1 was designed to yield the 323 bp band for *C. crispus*, the 421 bp band for other *Carduus*, and the 700 bp band for all examined samples (Fig. S3). By contrast, the combination of matK_463F, matK_1162R, CD_SNP_F2, and CD_SNP_R2 yielded a 421 bp PCR product for *C. crispus* and a 323 bp band for other *Carduus* species (Fig. S3). Reactions were conducted in 25 µl solution consisting of 50 ng of template DNA, 2.5 µl of 10× reaction buffer, 0.5 U of E-taq DNA polymerase, 50 mM MgCl_2_, and 5 mM dNTPs. Concentrations of outer primer pairs (matK_463F and matK_1162R) and inner primer pairs (CD_SNP_F1 and CD_SNP_R1, CD_SNP_F2 and CD_SNP_R2) were 0.75 pM and 0.5 pM, respectively. The PCR procedure consisted of 1 min at 94 °C, followed by denaturing for 1 min at 94 °C, annealing for 40 s at 55 °C, an extension stage of 50 s at 72 °C, and an additional extension of 7 min at 72 °C.

**Table 2:** Features of chloroplast genomes, assembly information, and pairwise identity among three *Carduus* species and related taxa.

**Species**	*Carduus crispus* (MK652229)	*C. tenuiflorus* (MK652230)	*C. acanthoides* (MK652228)	*Cynara humilis* (KP299292)	*C. baetica* (KP842706)	*C. cornigera* (KP842707)	*C. cardunculus var. scolymus* (KP842708)	*C. cardunculus var sylvestris* (KP842721)	*Cirsium arvense* (KY562583)	*Helianthus annus* (NC007977)
**Total reads**	21,118,624	4,621,758	4,300,959	–	–	–	–	–	–	–
**Assemble read**	805,076 (3.8%)	189,215 (4.1%)	180,245 (4.2%)	–	–	–	–	–	–	–
**Coverage**	1,585	372	354	–	–	–	–	–	–	–
**Number of contigs**	126	119	14	–	–	–	–	–	–	–
**N50 value (bp)**	95,199	50,873	128,044	–	–	–	–	–	–	–
**Total length**	152,342	152,426	152,617	152,585	152,548	152,550	152,529	152,528	152,855	151,104
**LSC**	83,254	83,360	83,532	83,622	83,599	83,580	83,578	83,577	83,859	83,530
**SSC**	18,706	18,674	18,693	18,651	18,639	18,660	18,641	18,641	18,633	18,308
**IR**	25,191	25,196	25,196	25,156	25,155	25,155	25,155	25,155	25,182	24,633
**Protein-coding genes**	80	80	80	80	80	80	80	80	80	80
**tRNA**	30	30	30	30	30	30	30	30	30	30
**rRNA**	4	4	4	4	4	4	4	4	4	4
**LSC-IR junction**	*rps19* (60 bp)	*rps19* (60 bp)	*rps19* (61 bp)	*rps19* (60 bp)	*rps19* (60 bp)	*rps19* (60 bp)	*rps19* (60 bp)	*rps19* (60 bp)	*rps19* (60 bp)	*rps19* (101 bp)
**SSC-IR junction**	*ycf1* (565 bp)	*ycf1* (565 bp)	*ycf1* (568 bp)	*ycf1* (567 bp) (*ycf1-ndhF* overlap 17 bp)	*ycf1* (567 bp) (*ycf1-ndhF* overlap 17 bp)	*ycf1* (567 bp) (*ycf1-ndhF* overlap 17 bp)	*ycf1* (567 bp) (*ycf1-ndhF* overlap 17 bp)	*ycf1* (567 bp) (*ycf1-ndhF* overlap 17 bp)	*ycf1* (565 bp)	*ycf1* (576 bp)
**Pairwise identity (%)**										
*C. crispus*	100			98.5	98.5	98.5	98.5	98.5	98.8	92.9
*C. tenuiflorus*	99.6	100		98.5	98.5	98.5	98.5	98.5	98.9	92.3
*C. acanthoides*	99.2	99.3	100	98.7	98.6	98.7	98.7	98.7	98.7	92.3


**Notes.**

The dashes (–) mean no data in this study.

## Results

### Comparative chloroplast genome analysis of the focal species

**Figure 1:**
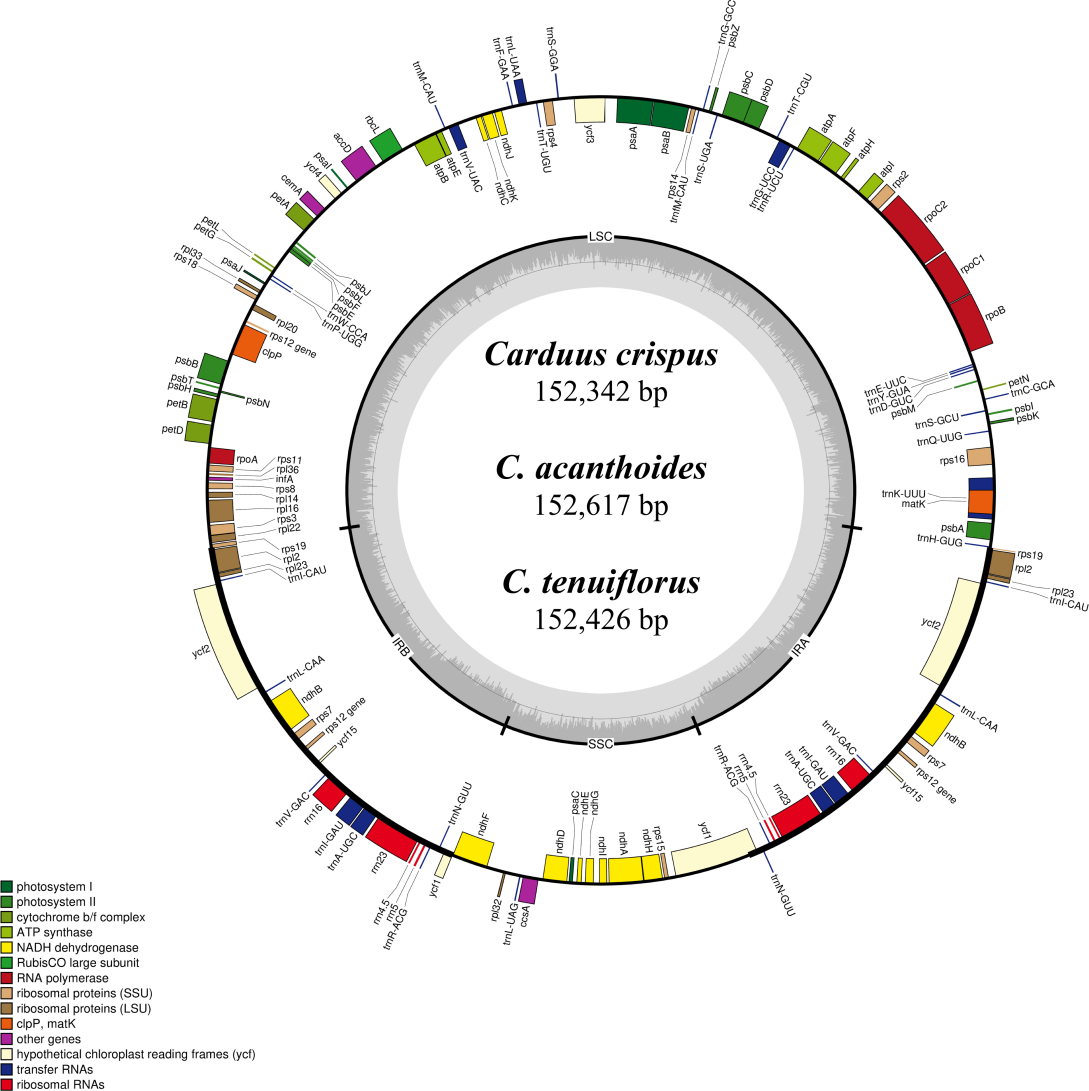
Map of the chloroplast genomes of *Carduus*. Genes inside the circle are transcribed clockwise whereas genes outside the circle are transcribed counterclockwise. LSC, large single copy; SSC, small single copy; IRA–IRB, inverted repeat regions.

**Figure 2:**
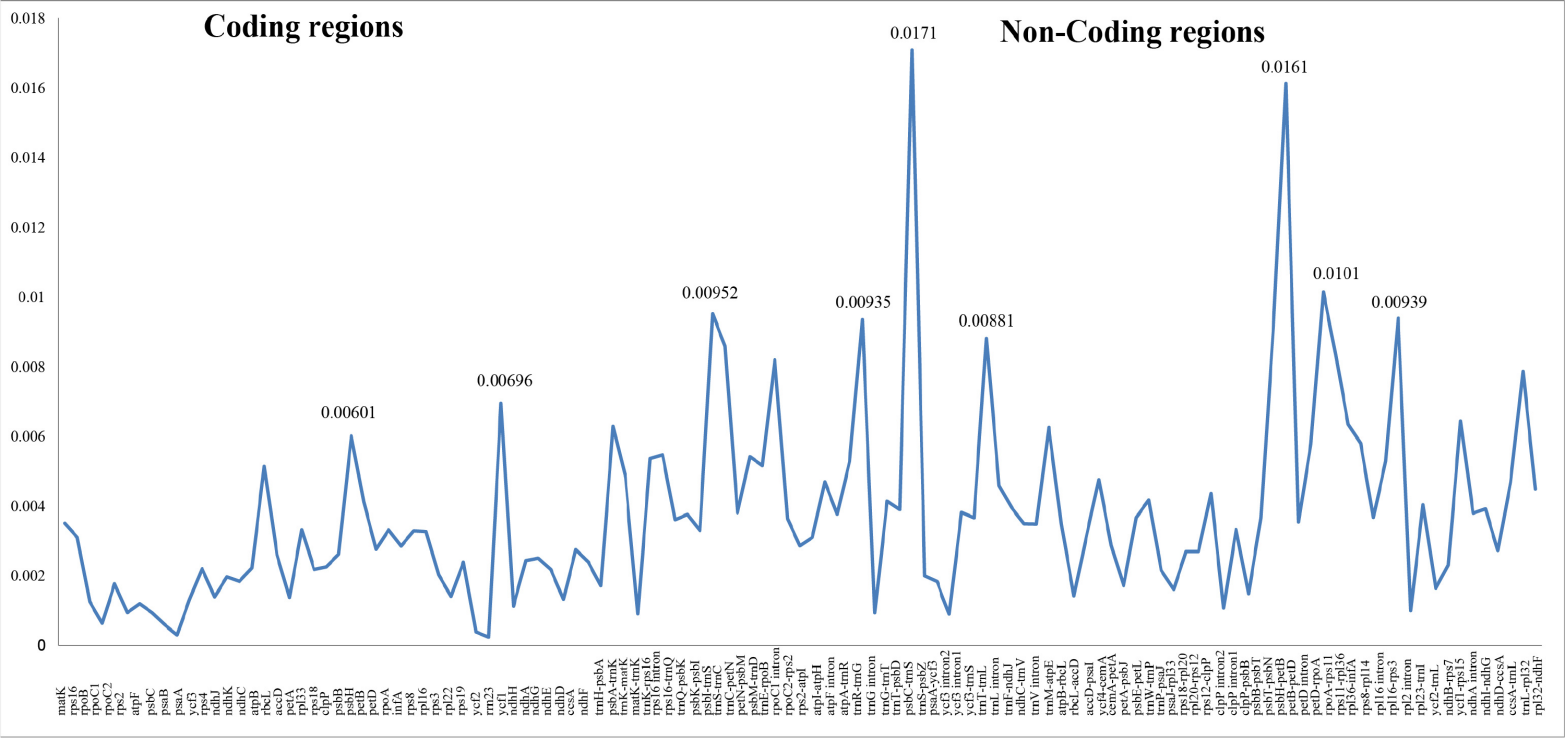
Nucleotide diversity (Pi values) among the three *Carduus* species.

Differing numbers of reads were obtained from NGS data, resulting in varying cpDNA coverage rates among the three focal species (Table 2). Total cpDNA length differed among species, ranging from 152,342 bp to 152,617 bp, and included a large single copy (LSC), a small single copy (SSC), and two IR regions (Fig. 1). By contrast, all three species had identical numbers of protein coding (80), tRNA (30), and rRNA (4) genes (Table 2, Table S3). The IR-LSC and IR-SSC junctions were located in the *rps19* and *ycf1* coding regions, respectively, but were longer in *C. acanthoides* (*rps19* = 61 bp and *ycf1* = 568 bp) than in the other two species (*rps19* = 60 bp and *ycf1* = 565 bp). In addition, pairwise identity indicated that *C. crispus* is more similar to *C. tenuiflorus* (99.6%) than *C. acanthoides* (99.2%). Observations of nucleotide diversity indicated that 119 of 131 surveyed regions differ among the three focal species (Table S2, Fig. 2). Compared to coding regions, non-coding sequences had higher Pi values (Fig. 2). The highest Pi values were found in the *psbC-trnS* (0.0171) and *psbH-petB* (0.0161) regions. The highest value in coding regions was 0.00696 for *ycf1* (Fig. 2). High nucleotide diversity regions (Pi values >0.008) included *psbI-trnS_GCU*, *trnE_UUC-rpoB*, *trnR_UCU-trnG_UCC*, *psbC-trnS_UGA*, *trnT_UGU-trnL_UAA*, *psbT-psbN*, *petD-rpoA*, and *rpl16-rps3*.

**Figure 3:**
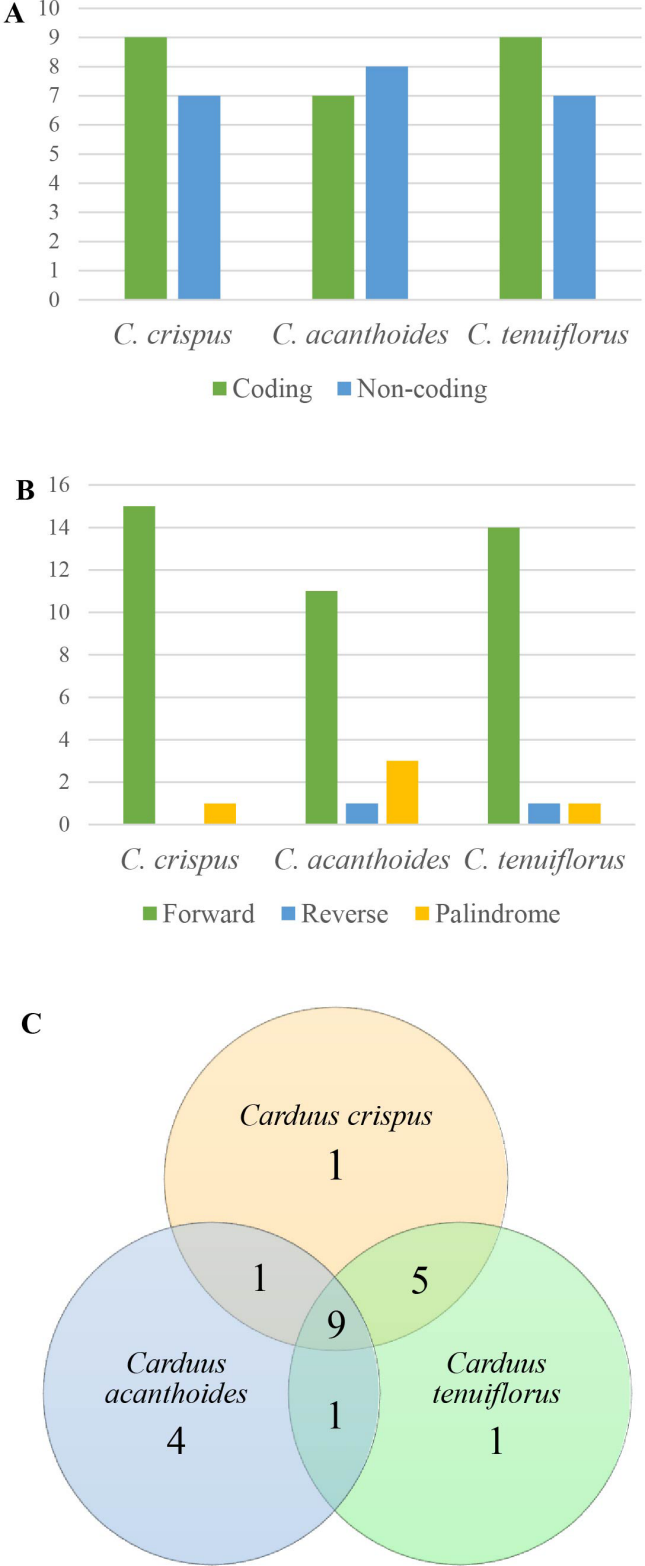
Repeats in the three *Carduus* species. (A) Distribution of repeats in the coding and non-coding regions, (B) composition of three types of repeat, and (C) unique and shared repeats in the three *Carduus* species.

### Features of cpDNA repeats

Analysis of SSRs yielded 43 SSRs in *C. crispus*, 40 in *C. tenuiflorus*, and 31 in *C. acanthoides* (Table S4). SSRs occupied the same position in all three species and were mostly located in non-coding regions. Although four types of SSR (i.e., mono-, di-, tri-, and tetra-nucleotides) were identified, most SSRs were mononucleotides composed of A and T nucleotides (Table S4). All 31 SSRs found in *C. acanthoides* were also present in the other two species (Table S4). By contrast, *C. crispus* had three unique SSRs and shared nine SSRs with *C. tenuiflorus*. There were no unique SSRs in *C. acanthoides* or specific shared SSRs between *C. acanthoides* and either *C. crispus* or *C. tenuiflorus*.

Among the focal species, 16 repeats were identified for both *C. crispus* and *C. tenuiflorus,* compared to 15 for *C. acanthoides* (Fig. 3A, Table S5). There were more repeats in coding regions than in non-coding areas, with the exception of *C. acanthoides*. Three types of repeats (i.e., forward, reverse, and palindrome) were identified in *C. tenuiflorus* and *C. acanthoides*; by contrast, only forward and palindrome repeats were found in *C. crispus*. Forward repeats were more abundant than reverse and palindrome repeats (Fig. 3B). Among recorded repeats, nine were common among all three species (Fig. 3C). *Carduus acanthoides* had four unique repeats, whereas *C. tenuiflorus* and *C. crispus* each had a single unique repeat. *Carduus acanthoides* shared one specific repeat with both *C. crispus* and *C. tenuiflorus*, whereas *C. crispus* and *C. tenuiflorus* shared five specific repeat regions (Fig. 3C).

### Phylogenetic relationships between *Carduus* and related taxa

**Figure 4:**
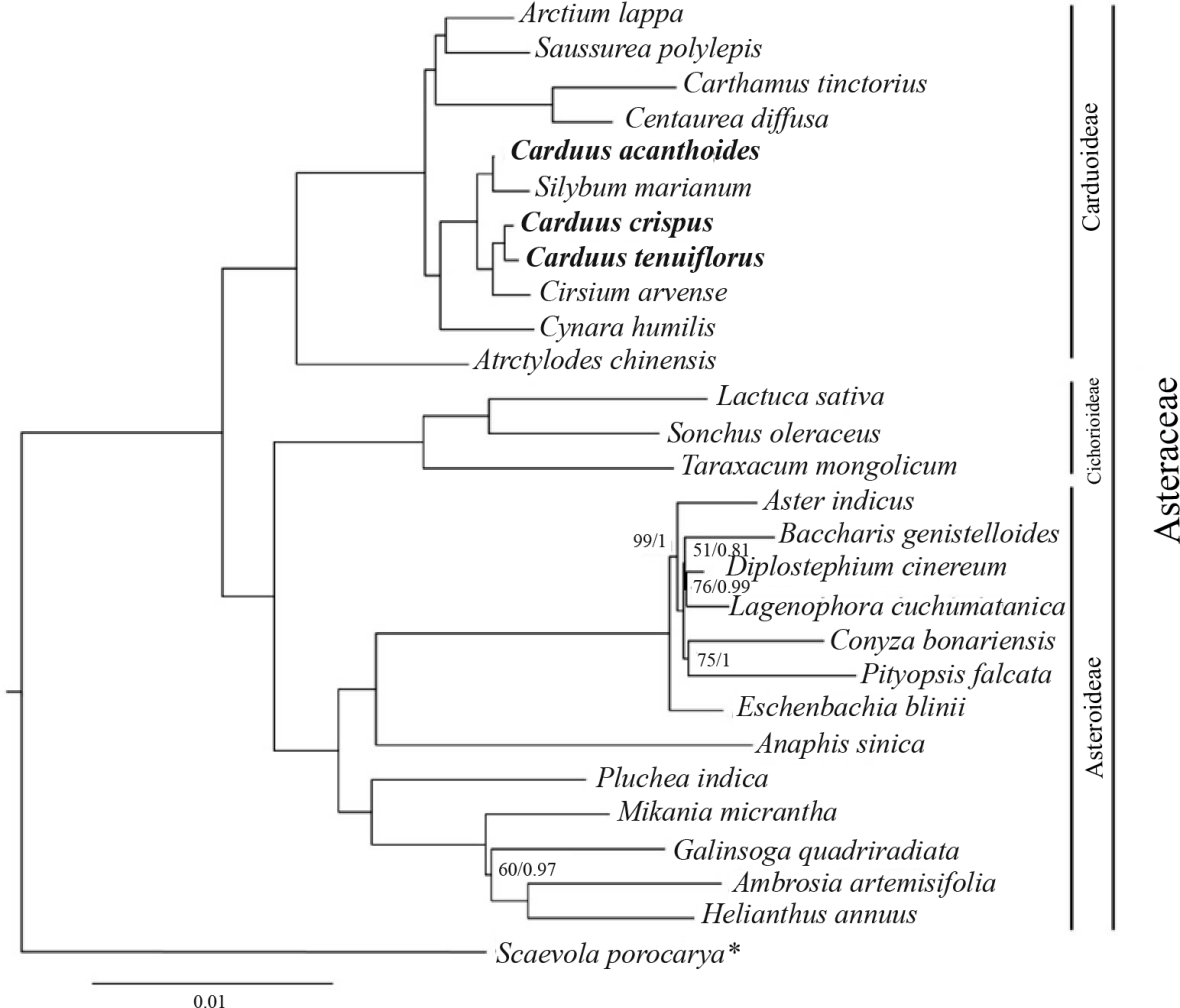
BI tree of *Carduus* and related taxa inferred from 78 protein coding cpDNA regions. Numbers indicate supporting values (BP, Bootstrap; PP, Posterior Probability). The asterisk indicates the *Scaevola porocarya* branch, which is compared with other species in the tree; this branch was reduced five times. Only supporting values under 100 (BP) or 1 (PP) are shown.

The ML and BI analyses, based on 78 protein-coding genes from *Carduus* and related taxa, yielded identical topologies (Fig. 4). In particular, both analyses confirmed the monophyly of Asteraceae subfamilies (i.e., Carduoideae, Chichorioideae, and Asteroideae). In contrast to the high support for Carduoideae and Cichorioideae (Bootstrap = 100/Posterior Probability = 1), low support was found for Asteroideae clades (Fig. 4). Notably, monophyly of the three *Carduus* species was not supported by either analysis. For example, *C. acanthoides* was sister to *Silybum marianum*, whereas *C. crispus* and *C. tenuiflorus* were sister to *Cirsium arvense*. Additional ML and BI analyses of full cpDNA sequences and non-coding regions suggested similar relationships (Fig. S4).

### Multiplex PCR and specific markers for *C. crispus*

**Figure 5:**
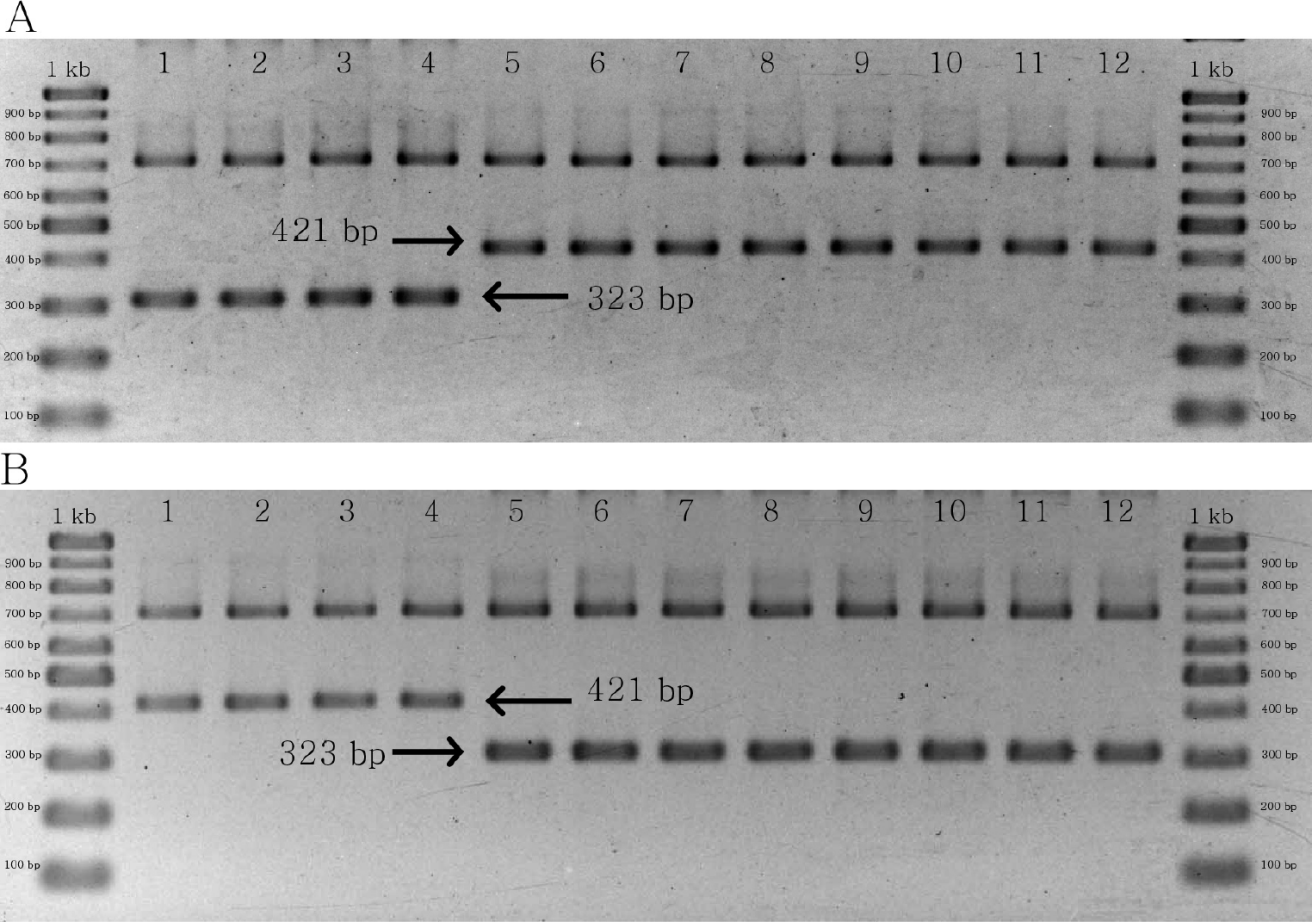
PCR results of specific primer pairs for *C. crispus*. (A) Combination of matK_463F, matK_1162R, CD_SNP_F1, and CD_SNP_R1, and (B) combination of matK_463F, matK_1162R, CD_SNP_F2, and CD_SNP_R2. Numbers 1–4 = *C. crispus*; 5–8 = *C. acanthoides*, and 9–12 = *C. tenuiflorus*.

The results of multiplex PCR for the two groups of primer pairs yielded similar products, both of which were designed to identify *C. crispus*. In the first group, a 323 bp band was found in *C. crispus,* whereas a 421 bp band was identified in *C. acanthoides* and *C. tenuiflorus* (Fig. 5A). By contrast, the combination of matK_463F, matK_1162R, CD_SNP_F2, and CD_SNP_R2 yielded a longer PCR product for *C. crispus* in comparison with the other species (Fig. 5B). The designed primer pairs were specific to all *C. crispus* samples examined in this study (Figs. S5 and S6).

## Discussion

### Conservatism of *Carduus* cpDNA

Chloroplast genomes are highly conserved in angiosperms with respect to gene content and order (Sugiura, 1992). This conservative tendency was observed in the three newly sequenced *Carduus* cpDNA genomes, compared to other Asteraceae members (Table 2). Other cpDNA sequences have revealed unique genomic events in Asteraceae. For example, the *atpB* gene, which encodes the CF1 ATPase beta subunit, is annotated as a pseudogene in *Aster spathulifolius* due to a deletion within the coding region (Choi and Park, 2015). Similarly, *trnT_GGU* was completely deleted or pseudogenised in the tribe Gnaphalieae (Lee et al., 2017). Duplication of *trnF_GAA* has been identified in *Taraxacum* (Salih et al., 2017). No comparable genomic events are present in *Carduus* or other members of subfamily Carduoideae (Table 2, Fig. S7). However, nucleotide diversity data pointed to potential regions for further study of phylogeny and population genetics, and the development of *Carduus*-specific molecular markers (Table S2, Fig. 2). The number of species we examined for this study was low relative to the approximately 32,000 known Asteraceae species. Therefore, additional studies that include the majority of Asteraceae species should be conducted to explore the overall evolutionary trends in the chloroplast genomes of this globally-distributed family.

Chloroplast genomes provide useful molecular data for reconstructing phylogeny, exploring biogeography, and estimating divergence time in angiosperm lineages (Do, Kim & Kim, 2014; Nguyen, Kim & Kim, 2015; Kim, Kim & Kim, 2016; Kim & Kim, 2018). Repetitive sequences in the chloroplast genome provide useful information for studying genomic rearrangement and phylogeny (Cavalier-Smith, 2002; Nie et al., 2012; Yi et al., 2013; Kim & Kim, 2018). In addition, existing repeats might result in the accumulation of new repeats in cpDNA (Asano et al., 2004). One of the crucial molecular data types in cpDNA is SSR sequences. Other studies have used SSRs to develop specific markers for different species and to study the genetic diversity of angiosperms (Ishikawa, Sakaguchi & Ito, 2016; Luo et al., 2016; Marochio et al., 2017; Han et al., 2018). In this study, although cpDNA sequences were highly conserved, the three *Carduus* species were found to have different numbers of SSRs (Table S4). While we did not develop SSR markers or conduct population studies of *Carduus,* the SSR information we obtained may be useful in future studies on *Carduus* species. In addition to the repeats shared among the three species, *C. crispus* had three unique repetitive sequences (Table S4), which may be useful in population studies, phylogenetic analyses, and the development of additional molecular markers.

### Uncertain relationships among *Carduus*

Phylogenetic analyses of the Asteraceae have identified ambiguous relationships between *Carduus*, *Cirsium*, and *Silybum* (Fu et al., 2016; Panero, 2016; Arnelas et al., 2018); for example, ITS data suggests that *Carduus* is polyphyletic (Häffner & Hellwig, 1999). Although three coding regions (*matK*, *rbcL*, and *ndhF*) were used to reconstruct phylogenetic relationships, the position of *Carduus* remained unresolved (Fu et al., 2016). We used 78 protein-coding regions to clarify these relationships; however, the phylogeny of *Carduus* and related taxa remains unclear (Fig. 4). Specifically, *C. acanthoides* was found to be close to *Silybum marianum* whereas *C. crispus* and *C. tenuiflorus* form a clade with *Cirsium arvense*. While non-coding regions can be useful in reconstructing the phylogeny of lower taxa, we were unable to recover the monophyly of *Carduus* using data from non-coding regions, including the eight hotspot areas as well as the combined data from coding and non-coding regions (Fig. S4). These issues suggest the need for additional studies on the phylogeny of *Carduus* and other members of the subfamily Carduoideae using supplementary molecular data and morphology.

### Implications of SNP data for developing molecular markers for *Carduus*

SNPs are useful in population studies due to its extremely abundant presence in the angiosperms genomes (Cui et al., 2017; Fischer et al., 2017; Pantoja et al., 2017), and are effective in phylogenetic analysis (Leaché & Oaks, 2017). In addition, various molecular markers have been developed for different angiosperm species based on SNP data from chloroplast genomes (Khlestkina & Salina, 2006; Wang et al., 2015a; Wang et al., 2015b; Hyun et al., 2019; Xia et al., 2019). We successfully developed a molecular marker, inferred from SNP data, to distinguish *C. crispus* from *C. acanthoides* and *C. tenuiflorus* (Fig. S3). Our marker demonstrates that nucleotide sequence variations can provide rapid molecular identification of *C. crispus*. We focused on *C. crispus* because it exhibits the characteristics of an invasive species (Dunn, 1976; Verloove, 2014; Jung et al., 2017), and may also have value for the treatment of obesity and cancer (Xie, Li & Jia, 2005; Davaakhuu, Sukhdolgor & Gereltu, 2010; Lee et al., 2011; Tunsag, Davaakhuu & Batsuren, 2011). Various DNA-based markers (i.e., inter-simple sequence repeats [ISSRs], sequence characterisation of amplified regions [SCARs], and SSRs) have been developed to authenticate medicinal plants to ensure safety and efficacy (Hao et al., 2010; Sarwat et al., 2012; Ganie et al., 2015; Ward, Gaskin & Wilson, 2008). Additionally, molecular data are useful for understanding invasion processes of alien plants (Ward et al., 2008). We developed a SNP-based molecular marker for *C. crispus* (Fig. 5, Figs. S5 and S6) that may be used to detect *C. crispus* invasions in their early stages, and to develop suitable management strategies. Our alignment results also identified specific SNPs for *C. acanthoides* and *C. tenuiflorus*, which may be used to create molecular markers for these species (Fig. S1). Although we only used the SNP in *matK*, use of the complete cpDNA sequences of *Carduus* will enable the mining of SNPs from other regions for developing molecular markers for *C. crispus* and related species.

## Conclusions

In this study, we provided the first complete cpDNA sequences for *Carduus* species. Despite the absence of significant differences (i.e., inversions, deletions, and duplications) between the chloroplast genomes of *Carduus* and those of related taxa, the newly acquired cpDNA sequences have value as a resource in future studies of the evolution of the chloroplast genome in Carduoideae and Asteraceae. Additionally, the 78 protein-coding regions of the chloroplast genome revealed uncertainty regarding the position of *Carduus* within the subfamily Carduoideae, and suggested the need for additional studies to reconstruct relationships not only among thistles, but among other members of the Asteraceae as well. The methods and protocols used in developing molecular markers for *C. crispus* are easy to apply and may be useful as a standard method in other studies of Asteraceae species.

##  Supplemental Information

10.7717/peerj.10687/supp-1 Supplemental Information 1 *Carduus acanthoides* chloroplast genome sequence

10.7717/peerj.10687/supp-2 Data S1 The GO annotation of *Carduus* species

10.7717/peerj.10687/supp-3 Supplemental Information 3 *Carduus crispuss* chloroplast genome sequence

10.7717/peerj.10687/supp-4 Supplemental Information 4 Supplemental tables and figures Table S1: List of species for phylogenomic analysis and whole cpDNA alignment. Table S2: List of regions for calculating Pi values. Table S3. Genes composition of the *Carduus* chloroplast genomes. Table S4: Feature of SSR in three *Carduus* species. Table S5: Feature of repeats in three *Carduus* species. Figure S1: The alignment of *matK* among three *Carduus*. The asterisk indicates the positions of single nucleotide polymorphism (SNP). The red square indicates SNP site for design primer pairs. Figure S2: The design of the primer pairs based on SNP site that is specific for *Carduus crispus*. Figure S3: The Bayesian Inference tree of *Carduus* and related taxa inferred from whole cpDNA sequences (A), non-coding regions of cpDNA (B), and eight hotspot regions (C).Figure S4: The PCR results of specific primer pairs for *Carduus crispus*. The combination of matK_463F, matK_1162R, CD_SNP_F1, and CD_SNP_R1. The number from 1 to 4: *Carduus crispus*; from 5 to 8: *Carduus acanthoides*; from 9 to 12: *Carduus tenuiflorus*. Figure S5: The PCR results of specific primer pairs for *Carduus crispus*. The combination of matK_463F, matK_1162R, CD_SNP_F2, and CD_SNP_R2. The number from 1 to 4: *Carduus crispus*; from 5 to 8: *Carduus acanthoides*; from 9 to 12: *Carduus tenuiflorus*. Figure S6: The MAUVE alignment of chloroplast genomes among *Carduus* and related species.

10.7717/peerj.10687/supp-5 Supplemental Information 5 *Carduus tenuiflorus* chloroplast genome sequence

## Additional Information and Declarations

### DNA Deposition

The following information was supplied regarding the deposition of DNA sequences: The complete cpDNA sequences of *C. crispus, C. acanthoides*, and *C. tenuiflorus* are available at GenBank: MK652229, MK652228, and MK652230, respectively.

### Data Availability

The following information was supplied regarding data availability: The three *Carduus* species’ sequence data are available at NCBI: PRJNA645567. Specimen voucher numbers and deposition information are available in Table 1.
