# Supplementary material for: Comparative analysis and implications of the chloroplast genomes of three thistles (Carduus L., Asteraceae)
Source: PeerJ. 2021 Jan 14;9:e10687. doi: 10.7717/peerj.10687 (PMC7811785; doi:10.7717/peerj.10687)
Supplement: Supplemental Information 4 — Table S1: List of species for phylogenomic analysis and whole cpDNA alignment. Table S2: List of regions for calculating Pi values. Table S3. Genes composition of the Carduus chloroplast genomes. Table S4: Feature of SSR in three Carduus species. Table S5: Feature of repeats in three Carduus species. Figure S1: The alignment of matK among three Carduus. The asterisk indicates the positions of single nucleotide polymorphism (SNP). The red square indicates SNP site for design primer pairs. Figure S2: The design of the primer pairs based on SNP site that is specific for Carduus crispus. Figure S3: The Bayesian Inference tree of Carduus and related taxa inferred from whole cpDNA sequences (A), non-coding regions of cpDNA (B), and eight hotspot regions (C).Figure S4: The PCR results of specific primer pairs for Carduus crispus. The combination of matK_463F, matK_1162R, CD_SNP_F1, and CD_SNP_R1. The number from 1 to 4: Carduus crispus; from 5 to 8: Carduus acanthoides; from 9 to 12: Carduus tenuiflorus. Figure S5: The PCR results of specific primer pairs for Carduus crispus. The combination of matK_463F, matK_1162R, CD_SNP_F2, and CD_SNP_R2. The number from 1 to 4: Carduus crispus; from 5 to 8: Carduus acanthoides; from 9 to 12: Carduus tenuiflorus. Figure S6: The MAUVE alignment of chloroplast genomes among Carduus and related species. [file peerj-09-10687-s004.docx]

Comparative analysis and implications of the chloroplast genomes of three thistles (*Carduus* L., Asteraceae)

**Joonhyung Jung**^1^, **Hoang Dang Khoa Do**^1,2^**, JongYoung Hyun**^1^**, ChangKyun Kim**^1^**, Joo‑Hwan Kim**^1^*

^1^ Department of Life Science, Gachon University, Seongnam, Republic of Korea 13120

^2^ Nguyen Tat Thanh Hi-Tech Institute, Nguyen Tat Thanh University, Ho Chi Minh City, Vietnam

**Author for correspondence:**

**Joo-Hwan Kim**

Department of Life Science, Gachon University, Seongnam, Republic of Korea 13120

Tell: +82317508827

Fax: +82317508738

Email: kimjh2009@gachon.ac.kr

**Supplementary Materials**:

The following are available online: **Table S1**: List of species for phylogenomic analysis and whole cpDNA alignment. **Table S2**: List of regions for calculating Pi values. **Table S3**. Genes composition of the *Carduus* chloroplast genomes. **Table S4**: Feature of SSR in three *Carduus* species. **Table S5**: Feature of repeats in three *Carduus* species. **Figure S1**: The alignment of *matK* among three *Carduus*. The asterisk indicates the positions of single nucleotide polymorphism (SNP). The red square indicates SNP site for design primer pairs. **Figure S2**: The alignment of partial *matK* sequences which include selected SNP among *Carduus* species. The light blue-shaded letter indicates the specific SNP of *C. crispus* in *matK*. **Figure S3**: The design of the primer pairs based on SNP site that is specific for *Carduus crispus*. The red bar indicates location of SNP in *matK*. The yellow arrows represent the position of primers CD_SNP_F2 and CD_SNP_R2 whereas the normal blue arrows means the location of primers CD_SNP_F1 and CD_SNP_R1. The bold blue arrows show the sites of matK463F and matK1162R. The two headed arrows indicate the size of each primer pairs: 700 bp for the pairs of matK463F and matK1162R, 421 bp for two pairs of matK463F-CD_SNP_R1 and matK463F-CD_SNP_R2, and 323 bp for two pairs of CD_SNP_F1-matK1162R and CD_SNP_F2-matK1162R. **Figure S4**: The Bayesian Inference tree of *Carduus* and related taxa inferred from whole cpDNA sequences (A), non-coding regions of cpDNA (B), and eight hotspot regions (C).**Figure S5**: The PCR results of specific primer pairs for *Carduus crispus*. The combination of matK_463F, matK_1162R, CD_SNP_F1, and CD_SNP_R1. The number from 1 to 12: *Carduus crispus*; from 13 to 20: *Carduus acanthoides*; from 21 to 24: *Carduus tenuiflorus*. **Figure S6**: The PCR results of specific primer pairs for *Carduus crispus*. The combination of matK_463F, matK_1162R, CD_SNP_F2, and CD_SNP_R2. The number from 1 to 12: *Carduus crispus*; from 13 to 20: *Carduus acanthoides*; from 21 to 24: *Carduus tenuiflorus*. **Figure S7**: The MAUVE alignment of chloroplast genomes among *Carduus* and related species.

Supplementary Table S1: List of species for phylogenomic analysis and whole cpDNA alignment

| Species | Accession number | Taxonomy |
| --- | --- | --- |
| *Scaevola porocarya** | KR604961 | Goodeniaceae |
| *Ambrosia artemisiifolia** | MG019037 | Asteraceae; Asteroideae; Heliantheae |
| *Anaphalis sinica** | KX148081 | Asteraceae; Asteroideae; Gnaphalieae |
| *Lactuca sativa** | AP007232 | Asteraceae; Cichorioideae; Cichorieae; Lactucinae |
| *Arctium lappa**§ | MH375874 | Asteraceae; Carduoideae; Cardueae; Carduinae |
| *Aster indicus ** | MG710386 | Asteraceae; Asteroideae; Astereae; Australasian lineages |
| *Atractylodes chinensis**§ | MG874805 | Asteraceae; Carduoideae; Cardueae; Carlininae |
| *Baccharis genistelloides** | KX063864 | Asteraceae; Asteroideae; Astereae; South American lineages |
| ***Carduus acanthoides******§ | MK652228 | Asteraceae; Carduoideae; Cardueae; Carduinae |
| ***Carduus crispus******§ | MK652229 | Asteraceae; Carduoideae; Cardueae; Carduinae |
| ***Carduus tenuiflorus******§ | MK652230 | Asteraceae; Carduoideae; Cardueae; Carduinae |
| *Carthamus tinctorius**§ | KP404628 | Asteraceae; Carduoideae; Cardueae; Centaureinae |
| *Centaurea diffusa**§ | KJ690264 | Asteraceae; Carduoideae; Cardueae; Centaureinae |
| ***Cirsium arvense******§ | KY562583 | Asteraceae; Carduoideae; Cardueae; Carduinae |
| *Conyza bonariensis** | MF276802 | Asteraceae; Asteroideae; Astereae; North American clade |
| ***Cynara humilis******§ | KP299292 | Asteraceae; Carduoideae; Cardueae; Carduinae |
| ***Cynara baetica*** | *KP842706* | Asteraceae; Carduoideae; Cardueae; Carduinae |
| ***Cynara cornigera*** | *KP842707* | Asteraceae; Carduoideae; Cardueae; Carduinae |
| ***Cynara cardunculus var. scolymus*** | *KP842708* | Asteraceae; Carduoideae; Cardueae; Carduinae |
| ***Cynara cardunculus var sylvestris*** | *KP842721* | Asteraceae; Carduoideae; Cardueae; Carduinae |
| *Diplostephium cinereum** | KX063889 | Asteraceae; Asteroideae; Astereae; South American lineages |
| *Eschenbachia blinii** | KX085421 | Asteraceae; Asteroideae; Astereae |
| *Galinsoga quadriradiata** | KX752097 | Asteraceae; Asteroideae; Heliantheae alliance; Millerieae |
| ***Helianthus annuus******§ | NC007977 | Asteraceae; Asteroideae; Heliantheae alliance; Heliantheae |
| *Silybum marianum**§ | KT267161 | Asteraceae; Carduoideae; Cardueae; Carduinae |
| *Taraxacum mongolicum** | KU736961 | Asteraceae; Cichorioideae; Cichorieae; Crepidinae |
| *Lagenophora cuchumatanica** | KX063879 | Asteraceae; Asteroideae; Astereae; Australasian lineages |
| *Sonchus oleraceus**§ | MG878405 | Asteraceae; Cichorioideae; Cichorieae; Hyoseridinae |
| *Saussurea polylepis**§ | MF695711 | Asteraceae; Carduoideae; Cardueae; Carduinae |
| ***Pluchea indica******§ | MG452144 | Asteraceae; Asteroideae; Inuleae; Plucheinae |
| *Mikania micrantha** | KX154571 | Asteraceae; Asteroideae; Heliantheae alliance; Eupatorieae |
| *Pityopsis falcata** | KY045817 | Asteraceae; Asteroideae; Astereae; North American clade |
| ***Lactuca sativa*** | NC_007578 | Asteraceae; Cichorioideae; Cichorieae; Lactucinae |
| ***Nicotiana tabacum*** | NC_001879 | Solanaceae;Nicotianoideae; Nicotianeae |

The bold names indicate the species for whole cpDNA alignment; The asterisks mean the taxa for phylogenomic analysis. The § symbol indicate the species for phylogenetic analysis inferred from non-coding regions.

Supplementary Table S2: The nucleotide diversity (Pi (π) value) of coding and non-coding regions among three *Carduus* species

| **No** | **Region** | **Length (bp)** | **Pi value** | **SD value** |
| --- | --- | --- | --- | --- |
| 1 | *trnH-psbA* | 388 | 0.00172 | 0.000081 |
| 2 | *psbA-trnK* | 222 | 0.00629 | 0.00296 |
| 3 | *trnK-matK* | 278 | 0.00483 | 0.00161 |
| 4 | *matK* | 1521 | 0.00351 | 0.00101 |
| 5 | *matK-trnK* | 735 | 0.00091 | 0.00043 |
| 6 | *trnK-rps16* | 784 | 0.00537 | 0.00146 |
| 7 | *rps16* | 215 | 0.0031 | 0.00146 |
| 8 | *rps16 intron* | 856 | 0.00547 | 0.00191 |
| 9 | *rps16-trnQ* | 941 | 0.00361 | 0.0014 |
| 10 | *trnQ-psbK* | 356 | 0.00376 | 0.00177 |
| 11 | *psbK-psbI* | 404 | 0.0033 | 0.0011 |
| 12 | *psbI-trn S* | 140 | 0.00952 | 0.00449 |
| 13 | *trn S-trnC* | 621 | 0.00859 | 0.00248 |
| 14 | *trnC-petN* | 537 | 0.00381 | 0.0018 |
| 15 | *petN-psbM* | 505 | 0.00542 | 0.00181 |
| 16 | *psbM-trnD* | 647 | 0.00516 | 0.00146 |
| 17 | *trnE-rpoB* | 872 | 0.0082 | 0.00346 |
| 18 | *rpoB* | 3183 | 0.00126 | 0.00037 |
| 19 | *rpoC1* | 2070 | 0.00064 | 0.0003 |
| 20 | *rpoC1 intron* | 732 | 0.00364 | 0.00136 |
| 21 | *rpoC2* | 4146 | 0.00177 | 0.00058 |
| 22 | *rpoC2-rps2* | 234 | 0.00286 | 0.00135 |
| 23 | *rps2* | 711 | 0.00094 | 0.00044 |
| 24 | *rps2-atpI* | 215 | 0.0031 | 0.00146 |
| 25 | *atpI-atpH* | 1139 | 0.0047 | 0.00196 |
| 26 | *atpF* | 555 | 0.0012 | 0.00057 |
| 27 | *atpF intron* | 707 | 0.00377 | 0.00178 |
| 28 | *atpA-trnR* | 126 | 0.00529 | 0.00249 |
| 29 | *trnR-trnG* | 323 | 0.00935 | 0.00441 |
| 30 | *trnG intron* | 706 | 0.00094 | 0.00045 |
| 31 | *trnG-trnT* | 161 | 0.00414 | 0.00195 |
| 32 | *trnT-psbD* | 1206 | 0.0039 | 0.0012 |
| 33 | *psbC* | 1422 | 0.00094 | 0.00044 |
| 34 | *psbC-trn S* | 234 | 0.01709 | 0.00806 |
| 35 | *trn S-psbZ* | 337 | 0.002 | 0.00094 |
| 36 | *psaB* | 2205 | 0.0006 | 0.0002 |
| 37 | *psaA* | 2253 | 0.0003 | 0.00014 |
| 38 | *psaA-ycf3* | 740 | 0.00183 | 0.00086 |
| 39 | *ycf3* | 507 | 0.00131 | 0.00062 |
| 40 | *ycf3 intron 2* | 742 | 0.0009 | 0.00042 |
| 41 | *ycf3 intron 1* | 698 | 0.00382 | 0.00142 |
| 42 | *ycf3-trn S* | 912 | 0.00366 | 0.00142 |
| 43 | *rps4* | 606 | 0.0022 | 0.00073 |
| 44 | *trnT-trnL* | 545 | 0.00881 | 0.00361 |
| 45 | *trnL intron* | 436 | 0.00459 | 0.00161 |
| 46 | *trnF-ndhJ* | 674 | 0.00396 | 0.00147 |
| 47 | *ndhJ* | 477 | 0.0014 | 0.00066 |
| 48 | *n dh K* | 678 | 0.00197 | 0.00093 |
| 49 | *ndhC* | 363 | 0.00184 | 0.00087 |
| 50 | *ndhC-trnV* | 1150 | 0.0035 | 0.0014 |
| 51 | *trnV intron* | 573 | 0.00349 | 0.00165 |
| 52 | *trnM-atpE* | 213 | 0.00626 | 0.00209 |
| 53 | *atpB* | 1494 | 0.00223 | 0.00087 |
| 54 | *atpB-rbcL* | 791 | 0.00349 | 0.00164 |
| 55 | *rbcL* | 1431 | 0.00514 | 0.00221 |
| 56 | *rbcL-accD* | 466 | 0.00143 | 0.00067 |
| 57 | *accD* | 1530 | 0.00261 | 0.00087 |
| 58 | *accD-psaI* | 669 | 0.00312 | 0.00147 |
| 59 | *ycf4-cemA* | 846 | 0.00476 | 0.00224 |
| 60 | *cemA-petA* | 233 | 0.00287 | 0.00135 |
| 61 | *petA* | 963 | 0.00138 | 0.00046 |
| 62 | *petA-psbJ* | 792 | 0.00172 | 0.00057 |
| 63 | *psbE-petL* | 1282 | 0.00367 | 0.00133 |
| 64 | *trnW-trnP* | 164 | 0.00417 | 0.00196 |
| 65 | *trnP-psaJ* | 308 | 0.00216 | 0.00102 |
| 66 | *psaJ-rpl33* | 430 | 0.00161 | 0.00076 |
| 67 | *rpl33* | 201 | 0.00332 | 0.00156 |
| 68 | *rps18* | 306 | 0.00218 | 0.00103 |
| 69 | *rps18-rpl20* | 248 | 0.0027 | 0.00127 |
| 70 | *rpl20-rps12* | 746 | 0.0027 | 0.00095 |
| 71 | *rps12-clpP* | 155 | 0.00436 | 0.00205 |
| 72 | *clpP* | 591 | 0.00226 | 0.00075 |
| 73 | *clpP intron 2* | 627 | 0.00108 | 0.00051 |
| 74 | *clpP intron 1* | 803 | 0.00333 | 0.00096 |
| 75 | *clpP-psbB* | 448 | 0.00149 | 0.0007 |
| 76 | *psbB* | 1527 | 0.00262 | 0.00105 |
| 77 | *psbB-psbT* | 184 | 0.00366 | 0.00173 |
| 78 | *psbT-psbN* | 73 | 0.00913 | 0.00431 |
| 79 | *psbH* | 222 | 0.00601 | 0.002 |
| 80 | *psbH-petB* | 124 | 0.01613 | 0.0076 |
| 81 | *petB* | 648 | 0.00412 | 0.00153 |
| 82 | *petB-petD* | 188 | 0.00355 | 0.00167 |
| 83 | *petD* | 483 | 0.00276 | 0.0013 |
| 84 | *petD intron* | 698 | 0.00579 | 0.00193 |
| 85 | *petD-rpoA* | 197 | 0.01015 | 0.00479 |
| 86 | *rpoA* | 1009 | 0.00332 | 0.00104 |
| 87 | *rpoA-rps11* | 80 | 0.00833 | 0.00393 |
| 88 | *rps11-rpl36* | 105 | 0.00635 | 0.00299 |
| 89 | *rpl36-infA* | 115 | 0.0058 | 0.00273 |
| 90 | *infA* | 234 | 0.00285 | 0.00134 |
| 91 | *rps8* | 405 | 0.00329 | 0.00155 |
| 92 | *rps8-rpl14* | 186 | 0.00368 | 0.00174 |
| 93 | *rpl16* | 408 | 0.00327 | 0.00154 |
| 94 | *rpl16 intron* | 1013 | 0.00529 | 0.00197 |
| 95 | *rpl16-rps3* | 149 | 0.00939 | 0.00313 |
| 96 | *rps3* | 657 | 0.00203 | 0.00068 |
| 97 | *rpl22* | 474 | 0.00141 | 0.00066 |
| 98 | *rps19* | 279 | 0.00239 | 0.00113 |
| 99 | *rpl2 intron* | 665 | 0.001 | 0.00047 |
| 100 | *rpl23-trnI* | 165 | 0.00404 | 0.0019 |
| 101 | *ycf2* | 6882 | 0.00039 | 0.00014 |
| 102 | *ycf2-trnL* | 408 | 0.00163 | 0.00077 |
| 103 | *ndhB-rps7* | 289 | 0.00231 | 0.00109 |
| 104 | *rrn23* | 2809 | 0.00024 | 0.00011 |
| 105 | *ycf1* | 5322 | 0.00696 | 0.00218 |
| 106 | *ycf1-rps15* | 433 | 0.00644 | 0.00304 |
| 107 | *ndhH* | 1182 | 0.00113 | 0.00053 |
| 108 | *ndhA* | 1092 | 0.00244 | 0.00115 |
| 109 | *ndhA intron* | 1082 | 0.00379 | 0.00111 |
| 110 | *ndhI-ndhG* | 340 | 0.00392 | 0.00185 |
| 111 | *ndhG* | 531 | 0.00251 | 0.00118 |
| 112 | *ndhE* | 306 | 0.00218 | 0.00103 |
| 113 | *ndhD* | 1503 | 0.00133 | 0.00036 |
| 114 | *ndhD-ccsA* | 246 | 0.00272 | 0.00128 |
| 115 | *ccsA* | 969 | 0.00275 | 0.00092 |
| 116 | *ccsA-trnL* | 148 | 0.00469 | 0.00221 |
| 117 | *trnL-rpl32* | 860 | 0.00787 | 0.00246 |
| 118 | *rpl32-ndhF* | 1051 | 0.00449 | 0.00163 |
| 119 | *ndhF* | 2235 | 0.00239 | 0.0008 |
| 120 | *atpF-atpA* | 66 | 0 | 0 |
| 121 | *psbZ-trnG* | 315 | 0 | 0 |
| 122 | *trnG-trnfM* | 186 | 0 | 0 |
| 123 | *rps4-trnT* | 372 | 0 | 0 |
| 124 | *trnL-trnF* | 365 | 0 | 0 |
| 125 | *psaI-ycf4* | 406 | 0 | 0 |
| 126 | *psbN-psbH* | 102 | 0 | 0 |
| 127 | *petB intron* | 772 | 0 | 0 |
| 128 | *ycf15-trnV* | 695 | 0 | 0 |
| 129 | *rrn5-trnR* | 251 | 0 | 0 |
| 130 | *ndhA-ndhI* | 101 | 0 | 0 |
| 131 | *ndhG-ndhE* | 222 | 0 | 0 |

Supplementary Table S3. Genes composition of the *Carduus* chloroplast genomes

| **Groups of genes** | **Names of genes** |
| --- | --- |
| Ribosomal RNAs | *rrn4*.*5*(*2x*), *rrn5*(*2x*), *rrn16*(*2x*), *rrn23*(*2x*) |
| Transfer RNAs | *trn****A****-UGC**(*2x*), *trn****C****-GCA*, *trn****D****-GUC*, *trn****E****-UUC*, *trn****F****-GAA*, *trn****G****_UCC*, trn****G****-GCC*, *trn****H****-GUG*(*2x*), *trn****I****-CAU*(*2x*),*trn****I****-GAU**(*2x*), *trn****K****-UUU**, *trn****L****-UAA**, *trn****L****-UAG*, *trn****L****-CAA*(*2x*), *trnf****M****-CAU*, *trn****M****-CAU*, *trn****N****-GUU*(*2x*), *trn****P****-UGG*, *trn****Q****-UUG*, *trn****R****-UCU*, *trn****R****-ACG*(*2x*),*trn****S****-GCU*, *trn****S****-UGA*, *trn****S****-GGA*, *trn****T****-GGU*, *trn****T****-UGU*, *trn****V****-UAC**, *trn****V****-GAC*(*2x*), *trn****W****-CCA*, *trn****Y****-GUA* |
| Photosystem I | *psaA*, *psaB*, *psaC*, *psaI*, *psaJ* |
| Photosystem II | *psbA*, *psbB*, *psbC*, *psbD*, *psbE*, *psbF*, *psbH*, *psbI*, *psbJ*, *psbK*, *psbL*, *psbM*,*psbN*, *psbT*, *psbZ* |
| Cytochrome | *petA*, *petB**, *petD**, *petG*, *petL*, *petN* |
| ATP synthases | *atpA*, *atpB*, *atpE*, *atpF**, *atpH*, *atpI* |
| Large unit of Rubisco | *rbcL* |
| NADH dehydrogenase | *ndhA**, *ndhB**(*2x*), *ndhC*, *ndhD*, *ndhE*, *ndhF*, *ndhG*, *ndhH*, *ndhI*, *ndhJ*, *ndhK* |
| ATP-dependent protease subunit P | *clpP** |
| Envelope membrane protein | *cemA* |
| Large units of ribosome | *rpl2**(*2x*), *rpl14*, *rpl16**, *rpl20*, *rpl22*, *rpl23*(*2x*), *rpl32*, *rpl33*, *rpl36* |
| Small units of ribosome | *rps2*, *rps3*, *rps4*, *rps7*(*2x*), *rps8*, *rps11*, *rps12**(*2x*), *rps14*, *rps15*, *rps16*, rps18*, *rps19* |
| RNA polymerase | *rpoA*, *rpoB*, *rpoC1**,*rpoC2* |
| Initiation factor | *infA* |
| Miscellaneous protein | *accD*, *ccsA*, *matK* |
| Hypothetical proteins and conserved reading frames | *ycf1*, *ycf2*(*2x*), *ycf3**, *ycf4*, *ycf15*(*2x*) |
| *- genes with introns; 2x-duplicated genes; ^Ψ^-pseudogenes. | |

| Supplementary Table S4: Feature of SSR in three *Carduus* species.   \| ***C. acanthoides*** \| \| \| \| \| \| \| --- \| --- \| --- \| --- \| --- \| --- \| \| **Types of repeat** \| **Sequence** \| **Start position** \| **End position** \| **Length** \| **Position in cpDNA** \| \| TTTC Repeat \| TTTCTTTCTTTC \| 81,725 \| 81,736 \| 12 \| *rpl16* intron \| \| GTTT Repeat \| GTTTGTTTGTTT \| 109,103 \| 109,114 \| 12 \| *ycf1* \| \| TTTC Repeat \| TTTCTTTCTTTC \| 116,647 \| 116,658 \| 12 \| *ndhA* intron \| \| AATC Repeat \| AATCAATCAATC \| 111,793 \| 111,804 \| 12 \| *ycf1* \| \| GATT Repeat \| GATTGATTGATT \| 116,240 \| 116,251 \| 12 \| *ndhA* intron \| \|  \|  \|  \|  \|  \|  \| \| TAA Repeat \| TAATAATAATAA \| 30,700 \| 30,711 \| 12 \| IGS (*trnT_CGU-psbD*) \| \| TTC Repeat \| TTCTTCTTCTTC \| 34,159 \| 34,170 \| 12 \| *psbC* \| \| AAT Repeat \| AATAATAATAAT \| 58,546 \| 58,557 \| 12 \| IGS (*accD-psaI*) \| \| GAA Repeat \| GAAGAAGAAGAA \| 108,950 \| 108,961 \| 12 \| *ycf1* \| \|  \|  \|  \|  \|  \|  \| \| TA Repeat \| TATATATATAT \| 18,238 \| 18,247 \| 10 \| *rpoC1* \| \| AT Repeat \| ATATATATATA \| 19,234 \| 19,243 \| 10 \| *rpoC2* \| \| AT Repeat \| ATATATATATA \| 26,183 \| 26,193 \| 10 \| *IGS (atpH-atpF)* \| \| TA Repeat \| TATATATATATA \| 67,324 \| 67,335 \| 12 \| IGS (*rpl33-rps18*) \| \|  \|  \|  \|  \|  \|  \| \| A Repeat \| AAAAAAAAAA \| 12,968 \| 12,977 \| 10 \| *rpoB* \| \| A Repeat \| AAAAAAAAAA \| 18,015 \| 18,024 \| 10 \| *rpoC1* \| \| T Repeat \| TTTTTTTTTT \| 25,625 \| 25,634 \| 10 \| IGS (*atpI-atpH*) \| \| A Repeat \| AAAAAAAAAA \| 77,274 \| 77,283 \| 10 \| IGS (*petD-rpoA*) \| \| T Repeat \| TTTTTTTTTT \| 58,890 \| 58,899 \| 10 \| IGS (*psaI-ycf4*) \| \| A Repeat \| AAAAAAAAAA \| 112,169 \| 112,178 \| 10 \| *ycf1* \| \| T Repeat \| TTTTTTTTTTTT \| 27,558 \| 27,569 \| 12 \| IGS (*atpF-atpA*) \| \| A Repeat \| AAAAAAAAAAAA \| 68,599 \| 68,610 \| 12 \| IGS (*rpl20-rps12*) \| \| T Repeat \| TTTTTTTTTTTTTTTT \| 79,952 \| 79,967 \| 16 \| IGS (*rps8-rpl14*) \| \| A Repeat \| AAAAAAAAAA \| 43,620 \| 43,629 \| 10 \| IGS (*ycf3-trnS_GGA*) \| \| A Repeat \| AAAAAAAAAAAAA \| 107,016 \| 107,026 \| 13 \| IGS (*trnR_ACG-rrn5S*) \| \| T Repeat \| TTTTTTTTTTT \| 62,755 \| 62,765 \| 11 \| IGS (*petA-psbJ*) \| \| A Repeat \| AAAAAAAAAAAAA \| 107,016 \| 107,028 \| 13 \| IGS (*ycf15-ycf1*) \| \| T Repeat \| TTTTTTTTTTT \| 1,969 \| 1,979 \| 11 \| *trnK_UUU* intron \| \| T Repeat \| TTTTTTTTTT \| 46,079 \| 46,088 \| 10 \| IGS (*trnT_UGU-trnL_UAA*) \| \| A Repeat \| AAAAAAAAAAAAAA \| 64,999 \| 65,012 \| 14 \| IGS (*psbE-petL*) \| \| T Repeat \| TTTTTTTTTTTTTTT \| 64,575 \| 64,589 \| 15 \| IGS (*psbE-petL*) \| \| T Repeat \| TTTTTTTTTT \| 77,520 \| 77,529 \| 10 \| *rpoA* \| \| ***C. crispus*** \| \| \| \| \| \| \| **Types of repeat** \| **Sequence** \| **Start position** \| **End position** \| **Length** \| **Position in cpDNA** \| \| AAAT Repeat \| AAATAAATAAAT \| 124,174 \| 124,188 \| 12 \| IGS (*rpl32-ndhF*) \| \| TTTC Repeat \| TTTCTTTCTTTCT \| 81,453 \| 81,465 \| 12 \| *rpl16* intron \| \| GTTT Repeat \| GTTTGTTTGTTT \| 108,823 \| 108,835 \| 12 \| *ycf1* \| \| TTTC Repeat \| TTTCTTTCTTTC \| 116,329 \| 116,341 \| 12 \| *ndhA* intron \| \| TTTC Repeat \| TTTCTTTCTTTCT \| 116,348 \| 116,360 \| 12 \| *ndhA* intron \| \| GATT Repeat \| AATCAATCAATC \| 111,507 \| 111,518 \| 12 \| *ycf1* \| \| GATT Repeat \| GATTGATTGATT \| 115,920 \| 115,931 \| 12 \| *ndhA* intron \| \|  \|  \|  \|  \|  \|  \| \| TAA Repeat \| TAATAATAATAA \| 30,469 \| 30,480 \| 12 \| IGS (*trnT_CGU-psbD*) \| \| TTC Repeat \| TTCTTCTTCTTC \| 33,937 \| 33,948 \| 12 \| *psbC* \| \| AAT Repeat \| AATAATAATAAT \| 58,287 \| 58,299 \| 12 \| IGS (*accD-psaI*) \| \| GAA Repeat \| GAAGAAGAAGAA \| 108,670 \| 108,681 \| 12 \| *ycf1* \| \|  \|  \|  \|  \|  \|  \| \| AT Repeat \| TATATATATA \| 18,114 \| 18,124 \| 10 \| *rpoC1* \| \| AT Repeat \| ATATATATAT \| 19,110 \| 19,120 \| 10 \| *rpoC2* \| \| AT Repeat \| ATATATATAT \| 26,061 \| 26,071 \| 10 \| *IGS (atpH-atpF)* \| \| AT Repeat \| TATATATATATA \| 67,070 \| 67,081 \| 12 \| IGS (*rpl33-rps18*) \| \|  \|  \|  \|  \|  \|  \| \| A Repeat \| TTTTTTTTTT \| 34,138 \| 34,147 \| 10 \| IGS (*psbC-trnS_UGA*) \| \| A Repeat \| TTTTTTTTTT \| 70,688 \| 70,697 \| 10 \| *clpP* intron \| \| A Repeat \| TTTTTTTTTT \| 12,844 \| 12,853 \| 10 \| *rpoB* \| \| A Repeat \| TTTTTTTTTT \| 17,891 \| 17,900 \| 10 \| *rpoC1* \| \| T Repeat \| TTTTTTTTTT \| 25,503 \| 25,512 \| 10 \| IGS (*atpI-atpH*) \| \| A Repeat \| AAAAAAAAAA \| 77,010 \| 77,019 \| 10 \| IGS (*petD-rpoA*) \| \| T Repeat \| TTTTTTTTTT \| 113,388 \| 113,397 \| 10 \| IGS (*ycf1-rps15*) \| \| T Repeat \| TTTTTTTTTTT \| 9,335 \| 9,345 \| 11 \| IGS (trnC_GCA-petN) \| \| C Repeat \| CCCCCCCCCCC \| 5,383 \| 5,393 \| 11 \| *rps16* intron \| \| A Repeat \| AAAAAAAAAAA \| 24,561 \| 24,571 \| 11 \| IGS (*atpI-atpH*) \| \| T Repeat \| TTTTTTTTTTT \| 73,211 \| 73,221 \| 11 \| IGS (*psbB-psbT*) \| \| A Repeat \| AAAAAAAAAAA \| 116,277 \| 116,287 \| 11 \| *ndhA* intron \| \| T Repeat \| TTTTTTTTTTT \| 58,625 \| 58,635 \| 11 \| IGS (*psaI-ycf4*) \| \| A Repeat \| AAAAAAAAAAA \| 111,882 \| 111,892 \| 11 \| *ycf1* \| \| T Repeat \| TTTTTTTTTTT \| 27,436 \| 27,446 \| 11 \| IGS (*atpF-atpA*) \| \| A Repeat \| AAAAAAAAAAA \| 68,345 \| 68,355 \| 11 \| IGS (*rpl20-rps12*) \| \| T Repeat \| TTTTTTTTTTT \| 79,685 \| 79,695 \| 11 \| IGS (*rps8-rpl14*) \| \| A Repeat \| AAAAAAAAAAAA \| 110,886 \| 110,897 \| 12 \| *ycf1* \| \| A Repeat \| AAAAAAAAAAAA \| 43,397 \| 43,408 \| 12 \| IGS (*ycf3-trnS_GGA*) \| \| A Repeat \| AAAAAAAAAAAA \| 106,737 \| 106,748 \| 12 \| IGS (*trnR_ACG-rrn5S*) \| \| T Repeat \| TTTTTTTTTTTT \| 62,501 \| 62,512 \| 12 \| IGS (*petA-psbJ*) \| \| A Repeat \| AAAAAAAAAAAA \| 106,737 \| 106,748 \| 12 \| IGS (*ycf15-ycf1*) \| \| A Repeat \| TTTTTTTTTTTTT \| 45,493 \| 45,505 \| 13 \| IGS (*rps4-ndhJ*) \| \| T Repeat \| TTTTTTTTTTTTT \| 1,970 \| 1,982 \| 13 \| *trnK_UUU* intron \| \| T Repeat \| TTTTTTTTTTTTTT \| 45,859 \| 45,872 \| 14 \| IGS (*trnT_UGU-trnL_UAA*) \| \| A Repeat \| AAAAAAAAAAAAAAA \| 64,755 \| 64,769 \| 15 \| IGS (*psbE-petL*) \| \| T Repeat \| TTTTTTTTTTTTTTTT \| 64,331 \| 64,346 \| 16 \| IGS (*psbE-petL*) \| \| T Repeat \| TTTTTTTTTTTTTTTTT \| 77,253 \| 77,269 \| 17 \| *rpoA* \| \| ***C. tenuiflorus*** \| \| \| \| \| \| \| **Types of repeat** \| **Sequence** \| **Start position** \| **End position** \| **Length** \| **Position in cpDNA** \| \| AAAT Repeat \| AAATAAATAAAT \| 124,253 \| 124,265 \| 12 \| IGS (*rpl32-ndhF*) \| \| TTTC Repeat \| TTTCTTTCTTTC \| 81,552 \| 81,563 \| 12 \| *rpl16* intron \| \| GTTT Repeat \| GTTTGTTTGTTT \| 108,934 \| 108,945 \| 12 \| *ycf1* \| \| TTTC Repeat \| TTTCTTTCTTTC \| 116,436 \| 116,447 \| 12 \| *ndhA* intron \| \| TTTC Repeat \| TTTCTTTCTTTC \| 116,455 \| 116,466 \| 12 \| *ndhA* intron \| \| GATT Repeat \| AATCAATCAATC \| 111,618 \| 111,629 \| 12 \| *ycf1* \| \| GATT Repeat \| GATTGATTGATT \| 116,030 \| 116,041 \| 12 \| *ndhA* intron \| \|  \|  \|  \|  \|  \|  \| \| TAA Repeat \| TAATAATAATAATAA \| 30,566 \| 30,580 \| 15 \| IGS (*trnT_CGU-psbD*) \| \| TTC Repeat \| TTCTTCTTCTTC \| 34,034 \| 34,045 \| 12 \| *psbC* \| \| AAT Repeat \| AATAATAATAAT \| 58,389 \| 58,401 \| 13 \| IGS (*accD-psaI*) \| \| GAA Repeat \| GAAGAAGAAGAA \| 108,781 \| 108,792 \| 12 \| *ycf1* \| \|  \|  \|  \|  \|  \|  \| \| AT Repeat \| TATATATATAT \| 18,190 \| 18,200 \| 10 \| *rpoC1* \| \| AT Repeat \| ATATATATATA \| 19,186 \| 19,196 \| 10 \| *rpoC2* \| \| AT Repeat \| ATATATATATA \| 26,140 \| 26,150 \| 10 \| *IGS (atpH-atpF)* \| \| AT Repeat \| TATATATATATA \| 67,159 \| 67,170 \| 12 \| IGS (*rpl33-rps18*) \| \|  \|  \|  \|  \|  \|  \| \| A Repeat \| TTTTTTTTTT \| 34,235 \| 34,244 \| 10 \| IGS (*psbC-trnS_UGA*) \| \| A Repeat \| TTTTTTTTTT \| 70,781 \| 70,790 \| 10 \| *clpP* intron \| \| A Repeat \| AAAAAAAAAA \| 12,920 \| 12,929 \| 10 \| *rpoB* \| \| A Repeat \| AAAAAAAAAA \| 17,967 \| 17,976 \| 10 \| *rpoC1* \| \| T Repeat \| TTTTTTTTTT \| 25,582 \| 25,591 \| 10 \| IGS (*atpI-atpH*) \| \| A Repeat \| AAAAAAAAAA \| 77,102 \| 77,111 \| 10 \| IGS (*petD-rpoA*) \| \| C Repeat \| CCCCCCCCCC \| 5,355 \| 5,364 \| 10 \| *rps16* intron \| \| A Repeat \| AAAAAAAAAAAAA \| 24,638 \| 24,650 \| 13 \| IGS (*atpI-atpH*) \| \| T Repeat \| TTTTTTTTTT \| 73,304 \| 73,313 \| 10 \| IGS (*psbB-psbT*) \| \| T Repeat \| TTTTTTTTTT \| 58,727 \| 58,736 \| 10 \| IGS (*psaI-ycf4*) \| \| A Repeat \| AAAAAAAAAAA \| 111,993 \| 112,003 \| 11 \| *ycf1* \| \| T Repeat \| TTTTTTTTTT \| 27,515 \| 27,524 \| 10 \| IGS (*atpF-atpA*) \| \| A Repeat \| AAAAAAAAAA \| 68,433 \| 68,442 \| 10 \| IGS (*rpl20-rps12*) \| \| T Repeat \| TTTTTTTTTTTT \| 79,778 \| 79,789 \| 12 \| IGS (*rps8-rpl14*) \| \| A Repeat \| AAAAAAAAAAAA \| 110,997 \| 111,008 \| 12 \| *ycf1* \| \| A Repeat \| AAAAAAAAAAAA \| 43,495 \| 43,506 \| 12 \| IGS (*ycf3-trnS_GGA*) \| \| A Repeat \| AAAAAAAAAAAAA \| 106,847 \| 106,859 \| 13 \| IGS (*trnR_ACG-rrn5S*) \| \| T Repeat \| TTTTTTTTTTTT \| 62,602 \| 62,613 \| 12 \| IGS (*petA-psbJ*) \| \| A Repeat \| AAAAAAAAAAAAA \| 106,847 \| 106,859 \| 13 \| IGS (*ycf15-ycf1*) \| \| A Repeat \| TTTTTTTTTTT \| 45,591 \| 45,601 \| 11 \| IGS (*rps4-ndhJ*) \| \| T Repeat \| TTTTTTTTTTTTT \| 1,980 \| 1,992 \| 13 \| *trnK_UUU* intron \| \| T Repeat \| TTTTTTTTTTTTTT \| 45,954 \| 45,967 \| 14 \| IGS (*trnT_UGU-trnL_UAA*) \| \| A Repeat \| AAAAAAAAAAAAAA \| 64,854 \| 64,867 \| 14 \| IGS (*psbE-petL*) \| \| T Repeat \| TTTTTTTTTTTTTT \| 64,432 \| 64,445 \| 14 \| IGS (*psbE-petL*) \| \| T Repeat \| TTTTTTTTTTTTTTTTTT \| 77,345 \| 77,362 \| 18 \| *rpoA* \| |
| --- | --- | --- | --- | --- | --- | --- | --- | --- | --- | --- | --- | --- | --- | --- | --- | --- | --- | --- | --- | --- | --- | --- | --- | --- | --- | --- | --- | --- | --- | --- | --- | --- | --- | --- | --- | --- | --- | --- | --- | --- | --- | --- | --- | --- | --- | --- | --- | --- | --- | --- | --- | --- | --- | --- | --- | --- | --- | --- | --- | --- | --- | --- | --- | --- | --- | --- | --- | --- | --- | --- | --- | --- | --- | --- | --- | --- | --- | --- | --- | --- | --- | --- | --- | --- | --- | --- | --- | --- | --- | --- | --- | --- | --- | --- | --- | --- | --- | --- | --- | --- | --- | --- | --- | --- | --- | --- | --- | --- | --- | --- | --- | --- | --- | --- | --- | --- | --- | --- | --- | --- | --- | --- | --- | --- | --- | --- | --- | --- | --- | --- | --- | --- | --- | --- | --- | --- | --- | --- | --- | --- | --- | --- | --- | --- | --- | --- | --- | --- | --- | --- | --- | --- | --- | --- | --- | --- | --- | --- | --- | --- | --- | --- | --- | --- | --- | --- | --- | --- | --- | --- | --- | --- | --- | --- | --- | --- | --- | --- | --- | --- | --- | --- | --- | --- | --- | --- | --- | --- | --- | --- | --- | --- | --- | --- | --- | --- | --- | --- | --- | --- | --- | --- | --- | --- | --- | --- | --- | --- | --- | --- | --- | --- | --- | --- | --- | --- | --- | --- | --- | --- | --- | --- | --- | --- | --- | --- | --- | --- | --- | --- | --- | --- | --- | --- | --- | --- | --- | --- | --- | --- | --- | --- | --- | --- | --- | --- | --- | --- | --- | --- | --- | --- | --- | --- | --- | --- | --- | --- | --- | --- | --- | --- | --- | --- | --- | --- | --- | --- | --- | --- | --- | --- | --- | --- | --- | --- | --- | --- | --- | --- | --- | --- | --- | --- | --- | --- | --- | --- | --- | --- | --- | --- | --- | --- | --- | --- | --- | --- | --- | --- | --- | --- | --- | --- | --- | --- | --- | --- | --- | --- | --- | --- | --- | --- | --- | --- | --- | --- | --- | --- | --- | --- | --- | --- | --- | --- | --- | --- | --- | --- | --- | --- | --- | --- | --- | --- | --- | --- | --- | --- | --- | --- | --- | --- | --- | --- | --- | --- | --- | --- | --- | --- | --- | --- | --- | --- | --- | --- | --- | --- | --- | --- | --- | --- | --- | --- | --- | --- | --- | --- | --- | --- | --- | --- | --- | --- | --- | --- | --- | --- | --- | --- | --- | --- | --- | --- | --- | --- | --- | --- | --- | --- | --- | --- | --- | --- | --- | --- | --- | --- | --- | --- | --- | --- | --- | --- | --- | --- | --- | --- | --- | --- | --- | --- | --- | --- | --- | --- | --- | --- | --- | --- | --- | --- | --- | --- | --- | --- | --- | --- | --- | --- | --- | --- | --- | --- | --- | --- | --- | --- | --- | --- | --- | --- | --- | --- | --- | --- | --- | --- | --- | --- | --- | --- | --- | --- | --- | --- | --- | --- | --- | --- | --- | --- | --- | --- | --- | --- | --- | --- | --- | --- | --- | --- | --- | --- | --- | --- | --- | --- | --- | --- | --- | --- | --- | --- | --- | --- | --- | --- | --- | --- | --- | --- | --- | --- | --- | --- | --- | --- | --- | --- | --- | --- | --- | --- | --- | --- | --- | --- | --- | --- | --- | --- | --- | --- | --- | --- | --- | --- | --- | --- | --- | --- | --- | --- | --- | --- | --- | --- | --- | --- | --- | --- | --- | --- | --- | --- | --- | --- | --- | --- | --- | --- | --- | --- | --- | --- | --- | --- | --- | --- | --- | --- | --- | --- | --- | --- | --- | --- | --- | --- | --- | --- | --- | --- | --- | --- | --- | --- | --- | --- | --- | --- | --- | --- | --- | --- | --- | --- | --- | --- | --- | --- | --- | --- | --- | --- | --- | --- | --- | --- | --- | --- | --- | --- | --- | --- | --- | --- | --- | --- | --- | --- | --- | --- | --- | --- | --- | --- | --- | --- | --- | --- | --- | --- | --- | --- | --- | --- | --- | --- | --- | --- | --- | --- | --- | --- | --- | --- | --- | --- | --- | --- | --- | --- | --- | --- | --- | --- | --- | --- | --- | --- | --- | --- | --- | --- | --- | --- | --- | --- | --- | --- | --- | --- | --- | --- | --- | --- | --- | --- | --- | --- | --- | --- | --- | --- | --- | --- | --- | --- | --- | --- | --- | --- | --- | --- | --- | --- | --- | --- | --- | --- | --- | --- | --- | --- | --- | --- | --- | --- | --- | --- | --- | --- | --- | --- | --- | --- | --- | --- | --- | --- | --- | --- | --- | --- | --- | --- | --- | --- | --- | --- | --- | --- | --- | --- | --- | --- | --- | --- | --- | --- | --- | --- | --- | --- | --- | --- | --- | --- | --- | --- | --- | --- | --- | --- | --- | --- | --- | --- | --- | --- | --- | --- | --- | --- | --- | --- | --- | --- | --- | --- | --- | --- | --- | --- | --- | --- | --- | --- | --- | --- | --- | --- | --- | --- | --- | --- | --- | --- | --- | --- |

Supplementary Table S5: Feature of repeats in three *Carduus* species.

| Species | No | Type of repeat | Length | Sequence | Position |
| --- | --- | --- | --- | --- | --- |
| *Carduus acanthoides* | 1 | F | 45 | AAAGAATATAATTATAATAAAGAATAGAATTATAATAATTATAAT | *trnR-UCU-trnG-UCC IGS* |
|  | 2 | P | 48 | AATTGAAGTAATGAGCCTCCCAATATTGGGAGGCTCATTACTTCAATT | *psbT-psbN IGS* |
|  | 3 | F | 24 | GAAGAAGAGGATGAGGATGTAAAG | *ycf1* |
|  | 4 | P | 34 | TTTTTTTTATTTTTTAATTAAAAAATAAAAAAAA | *trnT-UGU-trnL-UAA IGS* |
|  | 5 | F | 34 | AAATACTTGACAAAAATAAAAAATATGAAGAAAA | *ycf1* |
|  | 6 | R | 28 | AATAATAAATATAAAATATAAATAATAA | *accD-psaI IGS* |
|  | 7 | P | 26 | GAAGCAGATGATTAATCATCTGCTTC | *ycf2* |
|  | 8 | F | 25 | TACAGAACCGTACATGAGATTTTCA | *ycf3 intron 1/rps12-ycf15 IGS/ndhA intron* |
|  | 9 | F | 24 | ACGATATTGATGCTAGTGACGATA | *ycf2* |
|  | 10 | F | 24 | AAGAGGATAGCAAGTTACAAATT | *psaJ-rpl33 IGS/ycf15-trnV-GAC IGS* |
|  | 11 | F | 23 | ATATGGGTGCTAGTTACGATAT | *ycf2* |
|  | 12 | F | 21 | AGGATGAGTATTTTTTTGGAA | *ycf1* |
|  | 13 | F | 21 | AGAGAGGGATTCGAACCCTCG | *trnS-GCU/ trnS-UGA/ trnS-GGA* |
|  | 14 | F | 21 | GACAGGATTTGAACCCGTGAC | *trnfM-CAU/trnP-UGG* |
|  | 15 | F | 20 | TATAATAAGATATCTTTATA | *accD-psaI IGS* |
|  |  |  |  |  |  |
| *Carduus crispus* | 1 | P | 48 | AATTGAAGTAATGAGCCTCCCAATATTGGGAGGCTCATTACTTCAATT | *psbT-psbN IGS* |
|  | 2 | F | 34 | AAATACTTGACAAAAATAAAAAATATGAAGAAAA | *ycf1* |
|  | 3 | F | 25 | TACAGAACCGTACATGAGATTTTCA | *ycf3 intron 1/rps12-ycf15 IGS/ndhA intron* |
|  | 4 | F | 24 | ACGATATTGATGCTAGTGACGATA | *ycf2* |
|  | 5 | F | 24 | AAGAGGATAGCAAGTTACAAATT | *psaJ-rpl33 IGS/ycf15-trnV-GAC IGS* |
|  | 6 | F | 23 | TGAGACTAAAAACAAAAATAATT | *ycf1* |
|  | 7 | F | 23 | CAAACAAGAGAAAGAAACAAGTA | *ndhA-ndhI IGS* |
|  | 8 | F | 23 | ATATGGGTGCTAGTTACGATAT | *ycf2* |
|  | 9 | F | 21 | AGAGAGGGATTCGAACCCTCG | *trnS-GCU/ trnS-UGA/ trnS-GGA* |
|  | 10 | F | 21 | GACAGGATTTGAACCCGTGAC | *trnfM-CAU/trnP-UGG* |
|  | 11 | F | 20 | TATAATAAGATATCTTTATA | *accD-psaI IGS* |
|  | 12 | F | 20 | GCTCTACCAACTGAGCTATA | *trnV-UAC/trnA-UGC* |
|  | 13 | F | 20 | AAAGAAAAATTTTTTAACAT | *ycf1/ trnL-UAG-rpl32 IGS* |
|  | 14 | F | 20 | TTTTTCTTTCTTTCTCTAT | *ndhA intron* |
|  | 15 | F | 21 | AGGATGAGTATTTTTTTGGAA | *ycf1* |
|  | 16 | F | 25 | ATTTGAGTTTGAGGCAATGGATACT | *rbcL* |
|  |  |  |  |  |  |
| *Carduus tenuiflorus* | 1 | P | 48 | AATTGAAGTAATGAGCCTCCCAATATTGGGAGGCTCATTACTTCAATT | *psbT-psbN IGS* |
|  | 2 | F | 34 | AAATACTTGACAAAAATAAAAAATATGAAGAAAA | *ycf1* |
|  | 3 | R | 26 | GAAGCAGATGATTAATCATCTGCTTC | *ycf2* |
|  | 4 | F | 26 | AGGATGAGTATTTTTTTGGAAAGCAA | *ycf1* |
|  | 5 | F | 25 | TACAGAACCGTACATGAGATTTTCA | *ycf3 intron 1/rps12-ycf15 IGS/ndhA intron* |
|  | 6 | F | 25 | ATTTGAGTTTGAGGCAATGGATACT | *rbcL* |
|  | 7 | F | 24 | ACGATATTGATGCTAGTGACGATA | *ycf2* |
|  | 8 | F | 24 | AAGAGGATAGCAAGTTACAAATT | *psaJ-rpl33 IGS/ycf15-trnV-GAC IGS* |
|  | 9 | F | 23 | TGAGACTAAAAACAAAAATAATT | *ycf1* |
|  | 10 | F | 23 | ATATGGGTGCTAGTTACGATAT | *ycf2* |
|  | 11 | F | 21 | AGAGAGGGATTCGAACCCTCG | *trnS-GCU/ trnS-UGA/ trnS-GGA* |
|  | 12 | F | 21 | GACAGGATTTGAACCCGTGAC | *trnfM-CAU/trnP-UGG* |
|  | 13 | F | 20 | TATAATAAGATATCTTTATA | *accD-psaI IGS* |
|  | 14 | F | 20 | AAAGAAAAATTTTTTAACAT | *ycf1/ trnL-UAG-rpl32 IGS* |
|  | 15 | F | 20 | GCTCTACCAACTGAGCTATA | *trnV-UAC/trnA-UGC* |
|  | 16 | F | 20 | TTTTTTCTTTCTTTCTCTAT | *ndhA intron* |


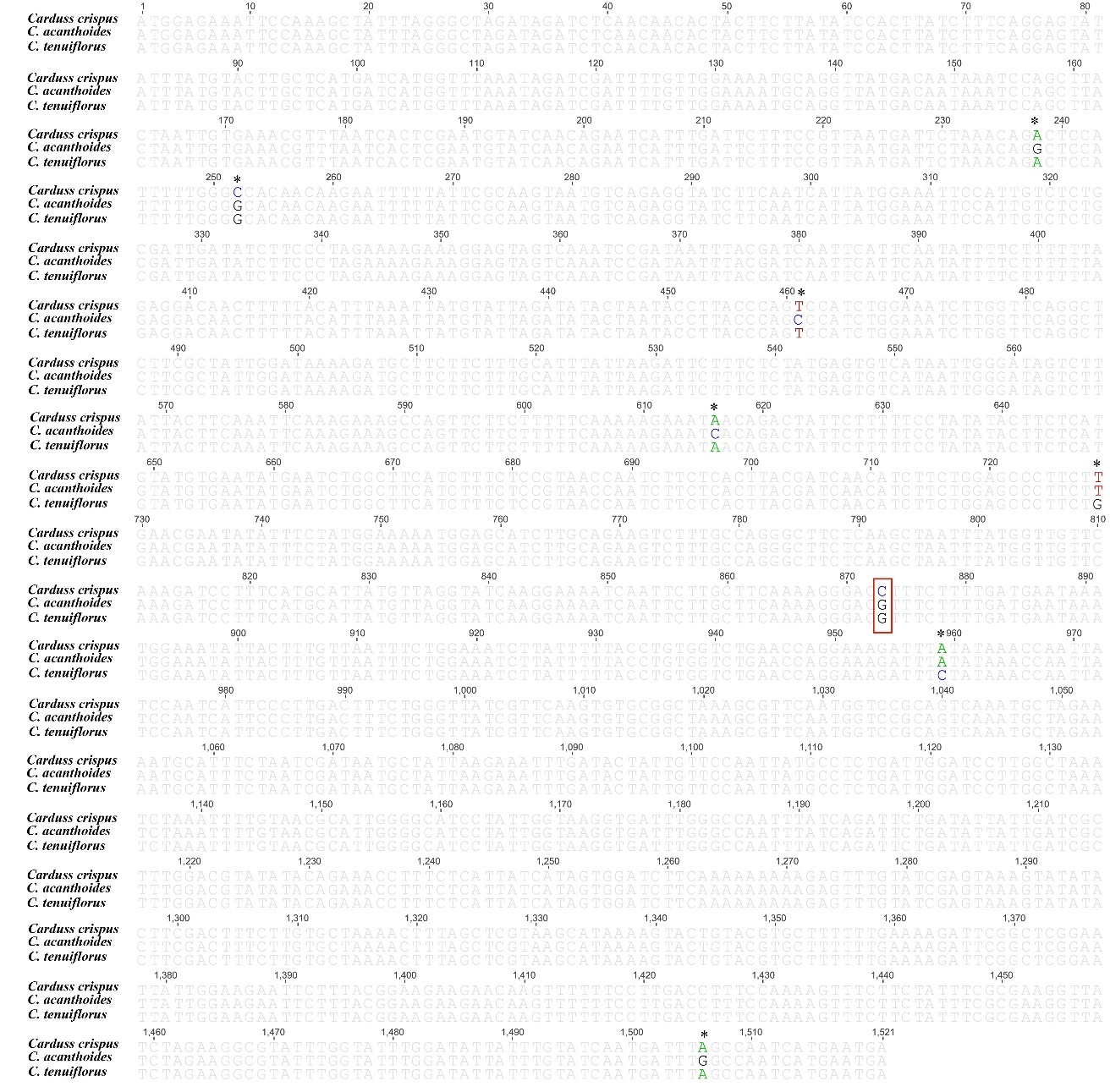


**Figure S1**: The alignment of *matK* among three *Carduus*. The asterisk indicates the positions of single nucleotide polymorphism (SNP). The red square indicates SNP site for design primer pairs.


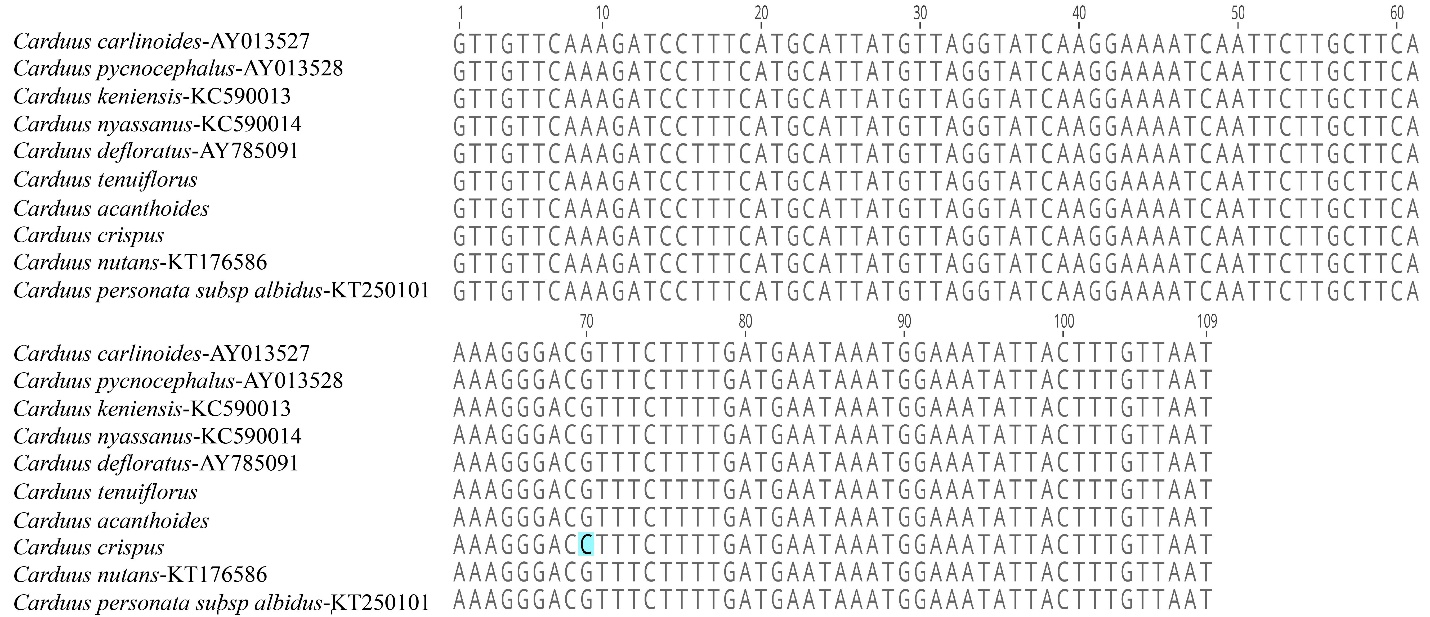


**Figure S2**: The alignment of partial *matK* sequences which include selected SNP among *Carduus* species. The light blue-shaded letter indicates the specific SNP of *C. crispus* in *matK*.


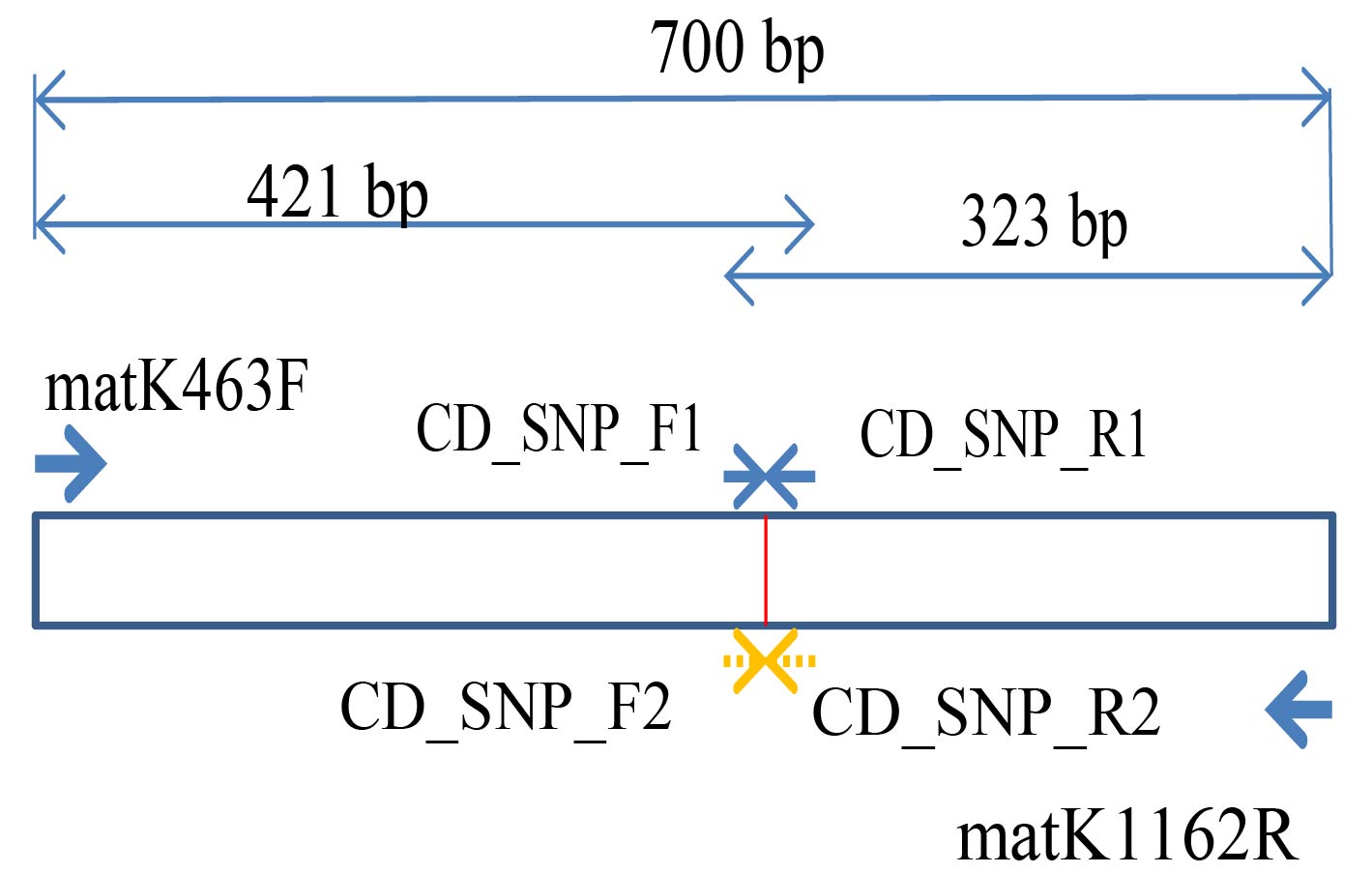


**Figure S3**: The design of the primer pairs based on SNP site that is specific for *Carduus crispus*. The red bar indicates location of SNP in *matK*. The yellow arrows represent the position of primers CD_SNP_F2 and CD_SNP_R2 whereas the normal blue arrows means the location of primers CD_SNP_F1 and CD_SNP_R1. The bold blue arrows show the sites of matK463F and matK1162R. The two headed arrows indicate the size of each primer pairs: 700 bp for the pairs of matK463F and matK1162R, 421 bp for two pairs of matK463F-CD_SNP_R1 and matK463F-CD_SNP_R2, and 323 bp for two pairs of CD_SNP_F1-matK1162R and CD_SNP_F2-matK1162R.


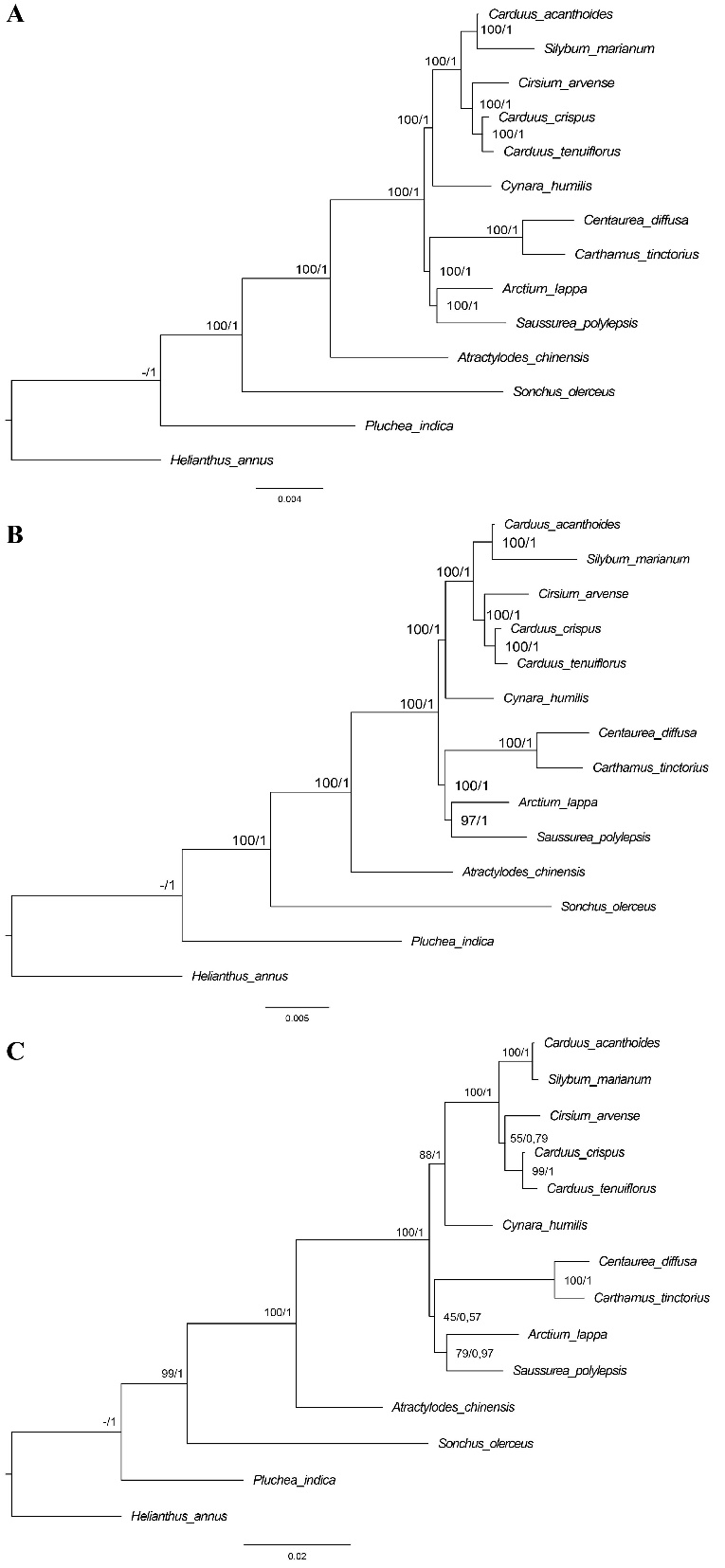


**Figure S4**: The Bayesian Inference tree of *Carduus* and related taxa inferred from whole cpDNA sequences (A), non-coding regions of cpDNA (B), and eight hotspot regions (C). The numbers mean supporting values (Bootstrap (BP)/ Posterior probability (PP)). The dash (-) means no support values.


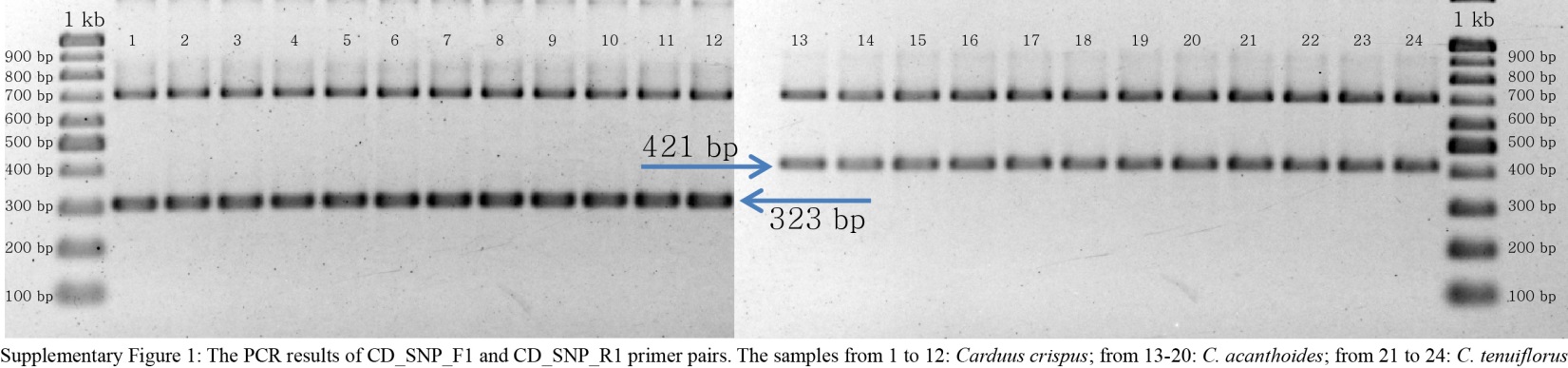


**Figure S5**: The PCR results of specific primer pairs for *Carduus crispus*. The combination of matK_463F, matK_1162R, CD_SNP_F1, and CD_SNP_R1. The number from 1 to 2: *Carduus crispus*; from 13 to 20: *Carduus acanthoides*; from 21 to 24: *Carduus tenuiflorus*.


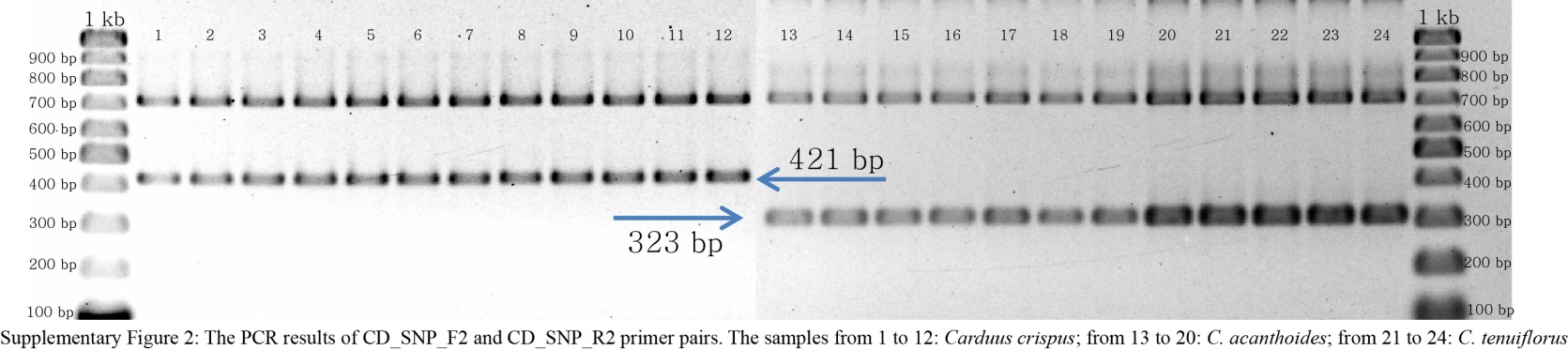


**Figure S6**: The PCR results of specific primer pairs for *Carduus crispus*. The combination of matK_463F, matK_1162R, CD_SNP_F2, and CD_SNP_R2. The number from 1 to 12: *Carduus crispus*; from 13 to 20: *Carduus acanthoides*; from 21 to 24: *Carduus tenuiflorus*.


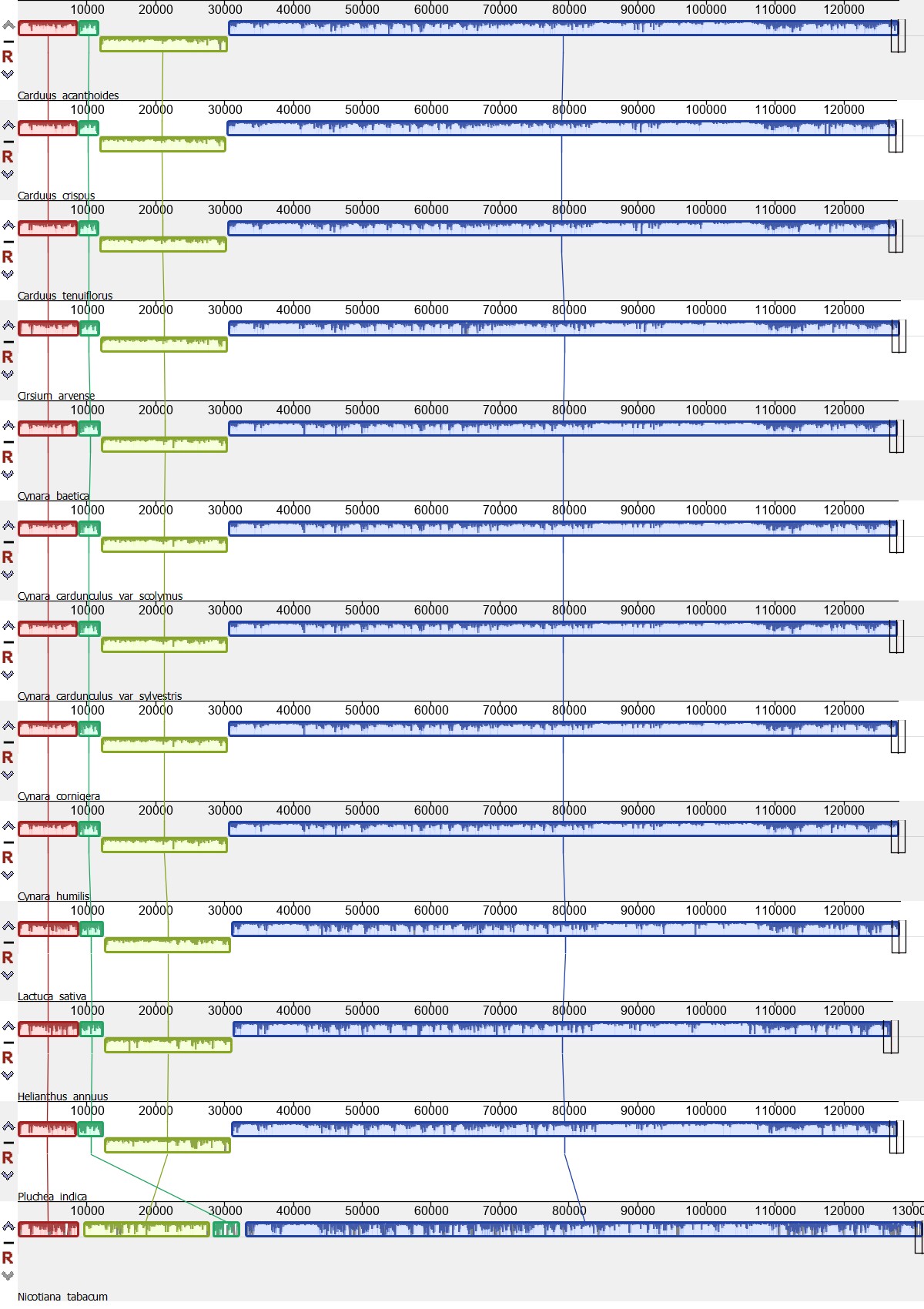


**Figure S7**: The MAUVE alignment of chloroplast genomes among *Carduus* and related species. *Nicotiana tabacum* is outgroup.
